# Enhanced FnCas12a-Mediated Targeted Mutagenesis Using crRNA With Altered Target Length in Rice

**DOI:** 10.3389/fgeed.2020.608563

**Published:** 2020-12-14

**Authors:** Katsuya Negishi, Masafumi Mikami, Seiichi Toki, Masaki Endo

**Affiliations:** ^1^Plant Genome Engineering Research Unit, Institute of Agrobiological Sciences, National Agriculture and Food Research Organization, Tsukuba, Japan; ^2^Graduate School of Nanobioscience, Yokohama City University, Yokohama, Japan; ^3^Kihara Institute for Biological Research, Yokohama City University, Yokohama, Japan; ^4^Probabilistic Modeling Team, Research Center for Agricultural Information Technology, National Agriculture and Food Research Organization, Tsukuba, Japan

**Keywords:** CRISPR/Cas12a, genome editing, targeted mutagenesis, large deletion, *Oryza sativa*

## Abstract

The CRISPR/Cas12a (Cpf1) system utilizes a thymidine-rich protospacer adjacent motif (PAM) and generates DNA ends with a 5′ overhang. These properties differ from those of CRISPR/Cas9, making Cas12a an attractive alternative in the CRISPR toolbox. However, genome editing efficiencies of Cas12a orthologs are generally lower than those of SpCas9 and depend on their target sequences. Here, we report that the efficiency of FnCas12a-mediated targeted mutagenesis varies depending on the length of the crRNA guide sequence. Generally, the crRNA of FnCas12a contains a 24-nt guide sequence; however, some target sites showed higher mutation frequency when using crRNA with an 18-nt or 30-nt guide sequence. We also show that a short crRNA containing an 18-nt guide sequence could induce large deletions compared with middle- (24-nt guide sequence) and long- (30-nt guide sequence) crRNAs. We demonstrate that alteration of crRNA guide sequence length does not change the rate of off-target mutation of FnCas12a. Our results indicate that efficiency and deletion size of FnCas12a-mediated targeted mutagenesis in rice can be fine-tuned using crRNAs with appropriate guide sequences.

## Introduction

The CRISPR/Cas9 (clustered regularly interspaced short palindromic repeats/CRISPR-associated protein 9) system was first reported as an adaptive immune system in archaea and bacteria and is now used for genome editing in various organisms, including plants (Li et al., 2013; Nekrasov et al., 2013; Shan et al., 2013). Cas9 endonuclease protein makes a complex with two small RNAs named CRISPR RNA (crRNA) and trans-activating crRNA (tracrRNA) (Jinek et al., 2012). The Cas9-RNA complex first recognizes a protospacer adjacent motif (PAM) sequence in the double-stranded DNA and then interrogates a target sequence next to the PAM (Shibata et al., 2017). Cas9 binds and cleaves target DNA with a sequence complementary to that of the crRNA to produce a DNA double-stranded break (DSB) that causes genome mutations as a failure of DNA repair pathways. In CRISPR/Cas9-mediated genome editing, the PAM restricts the selectivity of target sites because each Cas9 requires a specific PAM sequence for target recognition. The widely used Cas9 from *Streptococcus pyogenes* (SpCas9) recognizes an NGG sequence as a PAM. Cas9 orthologs from *Streptococcus thermophilus* (StCas9) and *Staphylococcus aureus* (SaCas9) recognize NNAGAA and NNGRRT as PAM sequences, respectively, and have been utilized for genome editing in plants (Steinert et al., 2015; Kaya et al., 2016). Furthermore, engineered SpCas9 variants that recognize different PAM sequences have been developed, expanding the application of genome editing in plants (Hu et al., 2018; Meng et al., 2018; Endo et al., 2019). These Cas9 orthologs and variants can expand target selectivity. However, Cas9 orthologs mainly require a guanine-rich sequence as a PAM. Cas12a—also known as CRISPR from *Prevotella* and *Francisella* 1 (Cpf1)—has been reported as another type of RNA-guided endonuclease derived from a Class 2/type V CRISPR/Cas system (Zetsche et al., 2015). While Cas9 requires a mainly G-rich sequence as a PAM, Cas12a can recognize a T-rich sequence as a PAM. Therefore, CRISPR/Cas12a-based genome editing technology can be a useful tool to complement CRISPR/Cas9 and further expand the targeting range. In addition, Cas12a has several features that differ from those of Cas9. Cas12a cleaves target DNA downstream of the PAM and produces cohesive ends with 5′ sticky overhangs, whereas Cas9 generates blunt ends upstream of the PAM (Zetsche et al., 2015). While Cas9 needs crRNA and tracrRNA, Cas12a requires only crRNA. The length of Cas12a crRNA is 40–45 nucleotides (nt), i.e., less than half the length of the SpCas9 single-guide RNA (sgRNA), which is a fusion RNA of crRNA and tracrRNA with an artificial linker (Jinek et al., 2012). Cas12a has both DNA and RNA cleavage activities to process the CRISPR precursor transcript (pre-crRNA) to mature crRNA, whereas Cas9 has DNA cleavage activity only (Fonfara et al., 2016). Three Cas12a orthologs, from *Acidaminococcus* sp. BV3L6 (AsCas12a), *Lachnospiraceae bacterium* ND2006 (LbCas12a), and *Francisella novicida* U112 (FnCas12a), have been used for genome editing in plants (Endo et al., 2016; Tang et al., 2017; Wang et al., 2017; Xu et al., 2019). AsCas12a and LbCas12a recognize TTTV and FnCas12a recognizes TTV as PAMs (Zetsche et al., 2015). However, mutagenesis efficiency using AsCas12a or LbCas12a was found to be generally lower than that using SpCas9 in maize (Lee et al., 2019). In our previous study of FnCas12a, the mutation efficiencies in several target sites designed in the *Nicotiana tabacum* genome were also very low—even below detection level (Endo et al., 2016). Because of the low mutation efficiency, Cas12a orthologs are thus harder to use for genome editing in plants than SpCas9 despite the many inherent advantages of Cas12a. Thus, the CRISPR/Cas12a system needs further optimization to improve genome editing efficiency. In SpCas9-mediated genome editing, there are several reports of enhancement of genome editing activity through gRNA engineering, such as changing the length of the sgRNA or scaffold sequence (Fu et al., 2014; Dang et al., 2015) or chemical modification of the sgRNA (Hendel et al., 2015; Ryan et al., 2018). In CRISPR/Cas12a, it has also been reported that engineering of the crRNA can affect genome editing activity. Modifications of the 3′-end sequence of crRNA can improve AsCas12a activity in human cells (Li et al., 2017). The FnCas12a-crRNA complex has DSB activity in *in vitro* assays when using crRNAs with 16- to 24-nt and 30-nt guide sequences (Lei et al., 2017). Although the most commonly used crRNAs of LbCas12a have a 25-nt guide and 21-nt scaffold sequence, LbCas12a can induce targeted mutations when using a crRNA containing a 31-nt guide, 21-nt scaffold, and 15-nt repeat spacer sequence in rice (Xu et al., 2017). Furthermore, the cleavage site recognized by FnCas12a could be altered by changing the crRNA length *in vitro*. The lengths of 5′ protruding ends were extended when the length of the guide sequence was 18-nt or less (Lei et al., 2017). In this work, we compared the mutation frequencies in rice using crRNAs with four different guide sequence lengths (18-nt, 24-nt, 30-nt, and 45-nt) and showed that the length of the guide sequence affects genome editing efficiency and mutation pattern. We also investigated the effect of guide sequence length on the rate of off-target mutation. Our results suggest that optimizing target length can lead to more efficient CRISPR/FnCas12a-mediated genome editing in plants.

## Materials and Methods

### Vector Construction

The FnCas12a vector used in this study is based on our previously described FnCas12a expression vectors, which include the FnCas12a expression cassette and the hygromycin B phosphotransferase (HPT) expression cassette (Endo et al., 2016). The crRNA of FnCas12a was placed under the control of the rice *U6-2* promoter (Mikami et al., 2015). crRNAs with 24-nt, 18-nt, 30-nt, or 45-nt guide sequences were inserted into the *Bbs*I site next to the crRNA scaffold. The expression cassette of crRNA was cloned into the binary vector using the restriction enzymes *Asc*I and *Pac*I (Endo et al., 2016).

### Transformation of Rice With FnCas12a/crRNA Expression Constructs

*Agrobacterium tumefaciens*-mediated transformation of rice (*Oryza sativa* L. cv. Nipponbare) using scutellum-derived calli was performed as described previously (Toki, 1997; Toki et al., 2006). Rice calli were infected by *A. tumefaciens* strain EHA105 transformed with the FnCas12a/crRNA vectors. Transgenic calli were selected for hygromycin resistance and cultured for 1 month at 30°C on callus induction medium containing 50 mg/L hygromycin B. Details of the rice transformation procedure have been described in a previous report (Mikami et al., 2017).

### Cleaved Amplified Polymorphic Sequences Analysis

To detect targeted mutations in the rice genome, genomic DNA was extracted from 18 to 25 independent transgenic calli or regenerated plants per construct using an Agencourt Chloropure Kit (Beckman Coulter). Target loci were amplified using the primers listed in Supplementary Table 1. PCR products were subjected to restriction enzyme digestion and analyzed by agarose gel electrophoresis. The number of samples for cleaved amplified polymorphic sequences (CAPS) analysis and the number of mutations detected in calli are shown in Supplementary Table 2.

### Sequencing Analysis

To determine mutation frequency in rice calli, we selected two representative lines for each construct whose CAPS analysis revealed a clear undigested PCR fragment, and their PCR products were cloned into pCR-BluntII-TOPO (Invitrogen) and subjected to sequence analysis using an Applied Biosystems 3500xl sequencer (Applied Biosystems).

### Amplicon Deep Sequencing Analysis

For amplicon deep sequencing analysis, the PCR products were adjusted in four steps: (1) in five target sites (DL-1, DL-2, ALS-1, ALS-2, and AAO2-1), crRNAs with short, middle, and long guide sequences were prepared and expressed with FnCas12a. Four independent transgenic calli with high mutation frequencies were selected by CAPS analysis. (2) Undigested PCR products indicating the occurrence of mutation were extracted using a DNA Gel Extraction Kit (QIAGEN) after agarose gel electrophoresis, and re-amplified to concentrate PCR products containing FnCas12a-mediated mutations. (3) PCR products derived from four independent calli were mixed in equal amounts. (4) Multiplex identifiers-labeled PCR products were sequenced on an Illumina MiSeq platform at FASMAC Co. (Japan). Mutations detected on fewer than 50 reads and at locations that were not around the target region were considered false positives due to PCR errors and were excluded from analysis. All primers for PCR are listed in Supplementary Table 1. The sequence data have been deposited with the DDBJ Sequence Read Archive (DRA) under accession number DRA010861.

## Results

### Effect of Guide Sequence Length on Mutation Frequency

**Figure 1:**
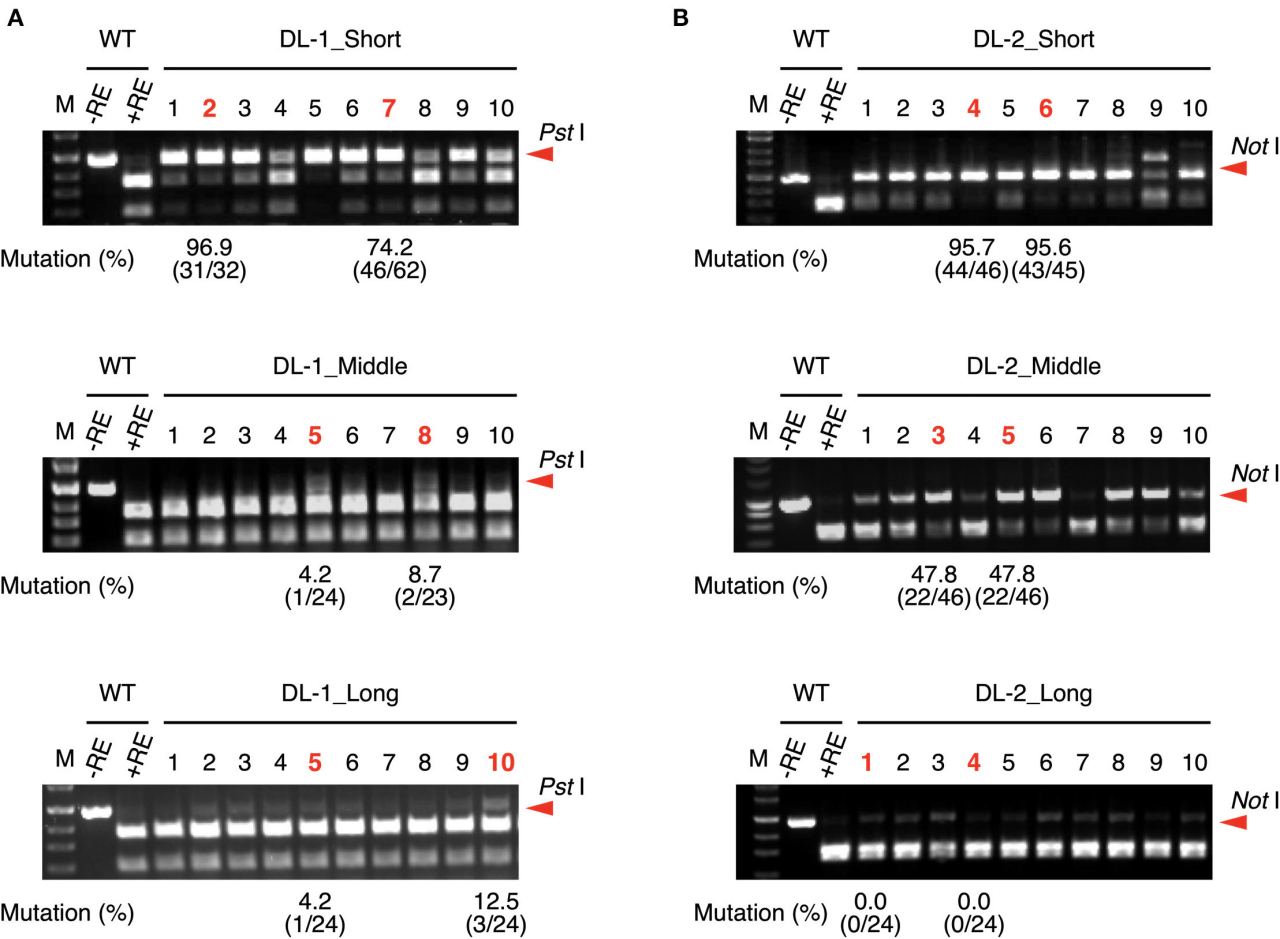
CAPS analysis and mutation frequency using crRNA with guide sequences of different lengths at DL-1 **(A)** and DL-2 **(B)** target sites. Mutation frequencies of selected calli (shown in red) were calculated from the ratio of sequenced clones with mutation. M, DNA molecular weight; –RE, without restriction enzyme; +RE, with restriction enzyme. Red arrowheads indicate the position of undigested PCR fragments.

The length of the crRNA of FnCas12a is generally 43-nt, comprising a 24-nt guide sequence that is complementary to the target DNA sequence and a 19-nt scaffold sequence (Zetsche et al., 2015). To investigate whether the length of guide sequence of crRNA affects targeted mutation efficiency in rice, we designed FnCas12a/crRNA vectors expressing crRNAs with 24-nt (middle), 18-nt (short), and 30-nt (long) guide sequences (Supplementary Figure 1). We selected two target sites in the rice *DROOPING LEAF* (*DL*) gene (Table 1). FnCas12a/crRNA vectors were transformed into rice calli via *A. tumefaciens* strain EHA105, and mutations were detected by CAPS analysis (Figure 1). In DL-1_Middle transformed calli, undigested DNA fragments, indicating the presence of mutation, were rarely detected (Figure 1A, middle panel). To estimate the mutation frequencies in independent transgenic calli, PCR products derived from calli lines #5 and #8 were cloned into plasmids and sequenced, showing that mutation frequencies in these lines were 4.1 and 8.3%, respectively (Figure 1A, middle panel). In contrast, when DL-1_Short was used, undigested DNA fragments were clearly detected in all transgenic calli, and mutation frequencies at the DL-1 target site were higher (up to 96.8% in callus line #2) than that of DL-1_Middle (Figure 1A, upper panel). The mutation frequency of DL-1_Long was comparable to that of DL-1_Middle (Figure 1A, lower panel). In the case of another target site, DL-2, the mutation frequencies of DL-2_Short were also higher than those of DL-2_Middle and DL-2_Long (Figure 1B). These results show that the use of crRNA with a shortened guide sequence at the DL-1 and DL-2 target sites could improve FnCas12a-mediated genome editing efficiency. To further investigate the effect of guide sequence length on mutation frequency, we selected additional eight target sites in five genes, *DL, ACETOLACTATE SYNTHASE* (*ALS*), *LOW CADMIUM* (*LCD*), *INDOLE-3-ACETALDEHYDE OXIDASE2* (*AAO2*), and *9-CIS-EPOXYCAROTENOID DIOXYGENASE1* (*NCED1*), and assessed their mutation frequencies (**Figure 4A**, Supplementary Figures 2–4, 9A). A summary of mutation frequency at each target site is shown in Table 2. In 4 out of 10 target sites (DL-1, DL-2, AAO2-1, and NCED1-1), using shortened guide sequences led to the highest mutation frequencies. On the other hand, in two target sites (ALS-1 and ALS-2), longer guide sequences improved mutation frequency compared with the middle guide sequence. For the other four target sites, the middle guide sequences showed the highest mutation frequencies, or we detected no mutations in all transgenic calli. These results suggest that FnCas12a-mediated mutation frequency could be improved by changing the length of the guide sequence. Previous *in vitro* experiments showed that FnCas12a could cleave the target DNA with crRNAs with a 16–30 nt guide (Zetsche et al., 2015; Lei et al., 2017), consistent with our *in vivo* results. We next investigated whether a guide sequence longer than 30 nt could further improve mutation frequencies *in vivo*. We designed four very-long-crRNAs with a 45-nt guide sequence at the *DL* gene (Supplementary Table 3). In CAPS assay, undigested DNA fragments were clearly detected in DL-2 and DL-3 target sites, meaning that very-long-crRNAs were functional in these target sites (Supplementary Figure 5). The mutation frequencies in DL-2 and DL-3 sites using very long guide sequences were 19.3 and 53.1%, respectively, i.e., slightly lower than frequencies achieved using middle guide sequences (Table 2). These results suggest that FnCas12a can work using crRNA with various lengths of guide sequence in plants.

**Table 1:** Target sequences and lengths of DL-1 and DL-2.

**Target gene**	**crRNA**	**PAM**	**Sequence**	**Length (nt)**
*DL*	DL-1_Short	TTC	GTCTTTTGGGTAGCTGCA	18
	DL-1_Middle		GTCTTTTGGGTAGCTGCAGGTTGG	24
	DL-1_Long		GTCTTTTGGGTAGCTGCAGGTTGGAGTCCC	30
	DL-2_Short	TTG	GGGAGAGCGGCTGCACCA	18
	DL-2_Middle		GGGAGAGCGGCTGCACCATCGGCG	24
	DL-2_Long		GGGAGAGCGGCTGCACCATCGGCGGCCGCG	30

*Underlined sequences indicate the restriction enzyme sites for CAPS assay*.

**Table 2:** Mutation frequencies using crRNA with different lengths of guide sequences.

**Target site**	**Mutation frequency (%)**				**Sequence**
	**Short (18 nt)**	**Middle (24 nt)**	**Long (30 nt)**	**Very long (45 nt)**	
DL-1	**81.9**	6.4	8.3	^*^	Table 1, Supplementary Table 3
DL-2	**95.6**	47.8	0.0	19.3	Table 1, Supplementary Table 3
DL-3	4.3	**85.4**	^*^	53.1	Supplementary Table 3
DL-4	8.2	**21.3**	^*^	^*^	Supplementary Table 3
ALS-1	20.0	46.8	**88.4**	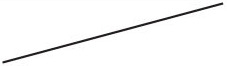	Supplementary Table 3
ALS-2	63.4	78.3	**93.5**	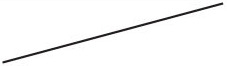	Supplementary Table 3
LCD-1	0.0	^*^	^*^	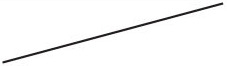	Supplementary Table 3
LCD-2	^*^	^*^	^*^	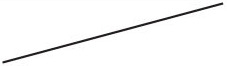	Table 3
AAO2-1	**75.8**	41.0	32.8	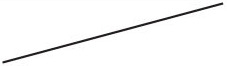	Supplementary Table 3
NCED1-1	**25.0**	20.0	18.8	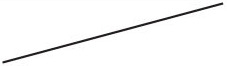	Supplementary Table 3

* *Undigested DNA fragments were not detected in all transgenic line in CAPS assay*.

*The highest mutation frequencies among the different guide lengths are shown in bold*.

### Analysis of Mutation Patterns Induced by Different Lengths of Guide Sequence

**Figure 2:**
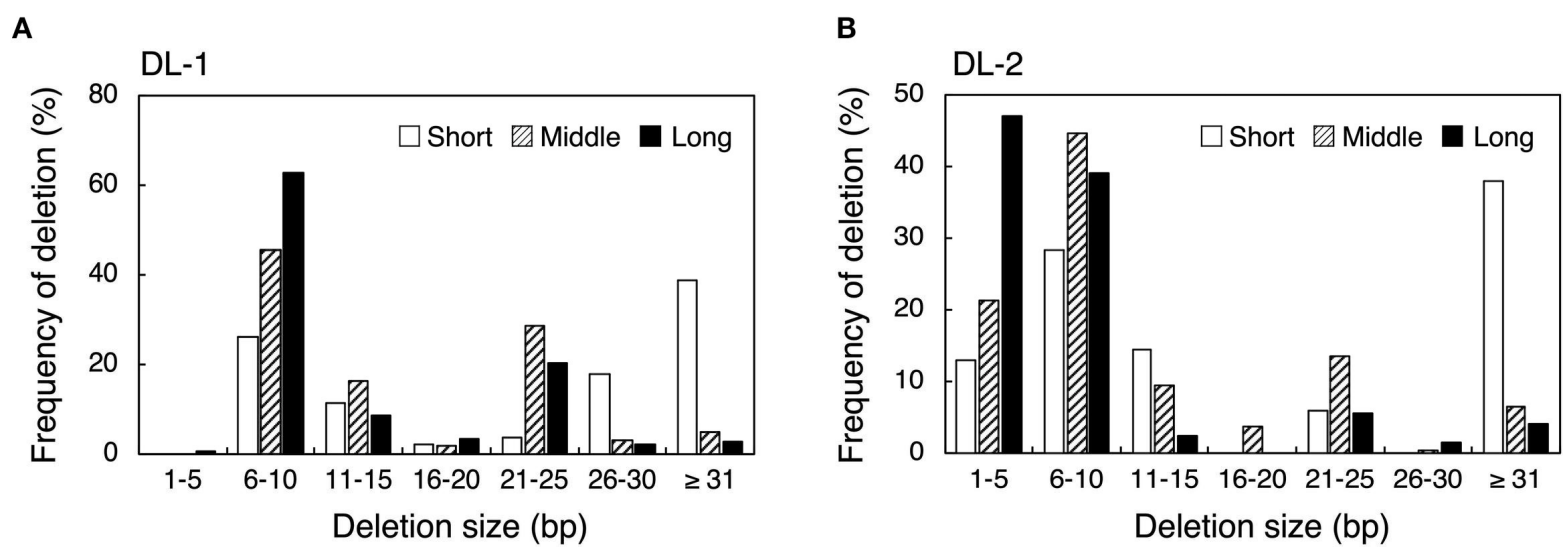
Comparison of deletion sizes using crRNA with guide sequences of different lengths at DL-1 **(A)** and DL-2 **(B)** target sites. Deletion size and mutation number were detected by targeted amplicon sequencing. Frequency of deletion means the percentage of deletions within the range of each deletion size per total number of deletion mutations.

**Figure 3:**
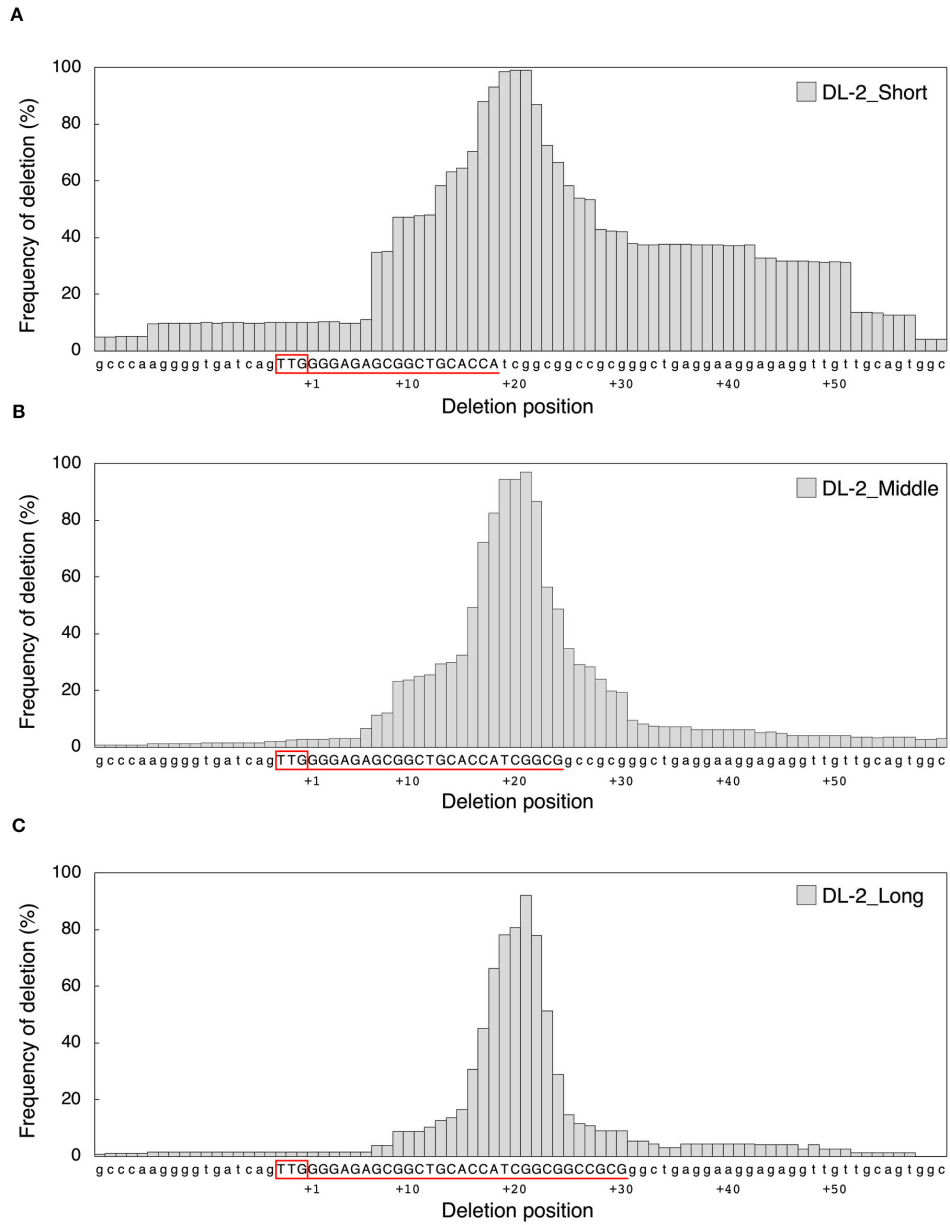
Comparison of deletion position according to the **(A)** DL2-Short, **(B)** DL-2_Middle, and **(C)** DL-2_Long guides at DL-2 target sites. Deletion size and mutation number were detected by targeted amplicon sequencing. Frequency of deletion means the percentage of deletions in each position among all deletion mutations. The target sequence of each guide is shown in red, underlined, and in capital letters. The PAM sequence is boxed in red upstream of the target sequence.

Next, we examined the effect of guide sequence length on mutation pattern. We investigated deletion size at DL-1 and DL-2 target sites by amplicon deep sequencing analysis (Figure 2). In DL-1_Middle and DL-1_Long, deletions of <31 bp accounted for more than 90%, and large deletions (≥31 bp) were rarely detected (Figure 2A, Supplementary Figure 7A). On the other hand, in DL-1_Short, large deletions were generated at a high frequency (38.9%) (Figure 2A). At the DL-2 target site, large deletions were also detected at high frequency in DL-2_Short (38.0%) compared with DL-2_Middle (6.6%) and DL-2_Long (4.1%) (Figure 2B, Supplementary Figure 7B). We also analyzed the deletion size in other target sites: ALS-1, ALS-2, and AAO2-1. Although the differences were less clear than in DL-1 and DL-2, the proportion of large deletions at these target sites also increased when using shorter guide sequences compared with middle and long guides (Supplementary Figures 6, 7). These results indicate that the use of short guide sequences tended to induce large deletions compared with those induced by middle and long guides. We next focused on the position of the deleted nucleotides. To investigate the frequency of deletion at each position of the target region, we collected deletion mutations from the NGS data and examined the frequency of deletion, which is the percentage of deletions at each position among all deletion mutations (Figure 3, Supplementary Figure 8). In DL-2 target sites, frequencies of deletion at 18–23 bp downstream of the PAM were >50% among the deletion mutations detected using all short, middle, and long guides (Figure 3A–C). In the case of the short guide, the frequency of deletion of nucleotides located at 24–51 bp downstream of PAM was ≥31% (Figure 3A). On the other hand, when using middle and long guides, the frequency of deletion in this region reduced gradually as the distance increased (Figures 3B,C). A similar result was obtained with DL-1 (Supplementary Figure 8). These results show that the large deletions detected using the short guide were due mainly to deletions in the region downstream of PAM.

### Off-Target Analysis Using Short and Long Guides

**Figure 4:**
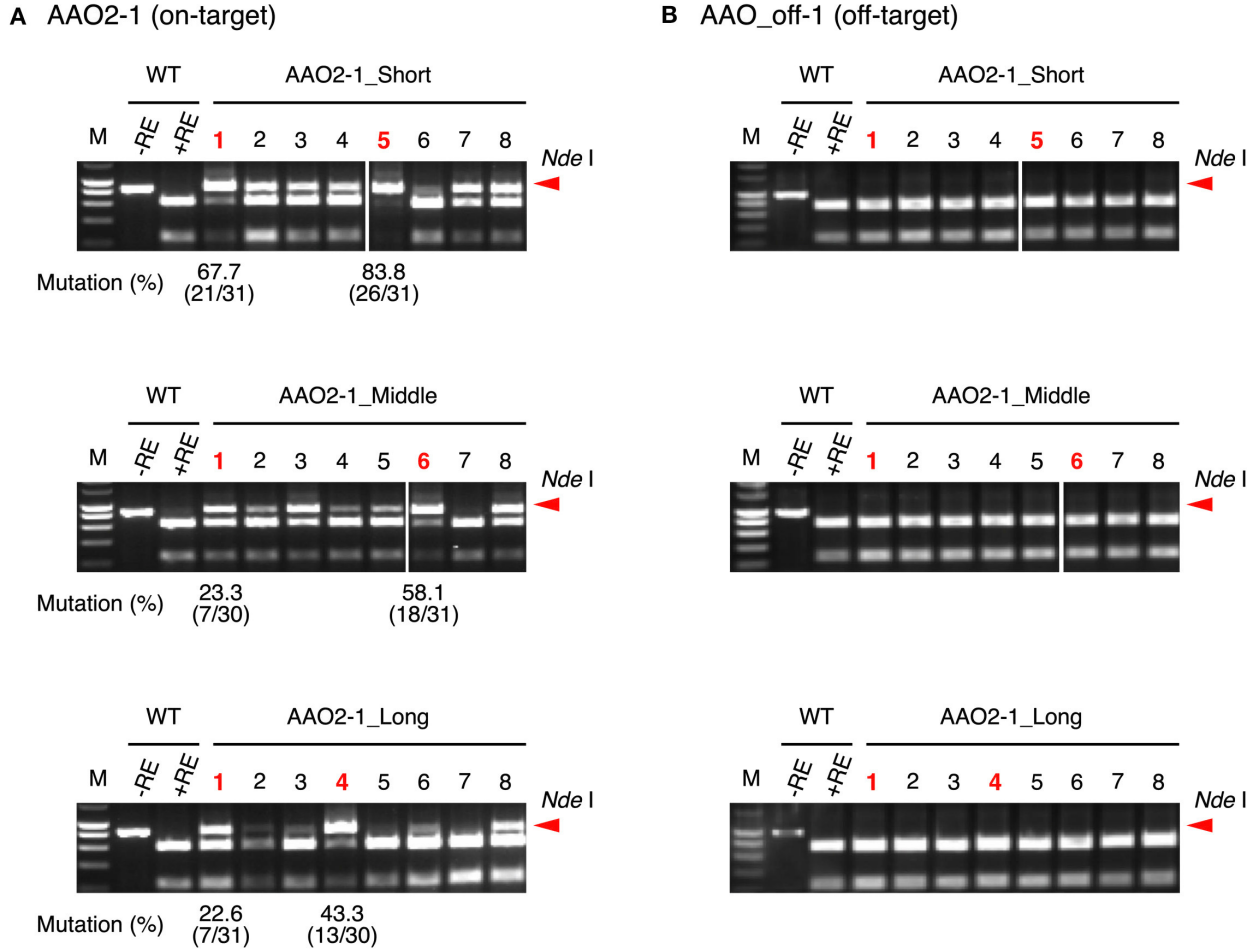
Off-target mutation analysis using CAPS assay at **(A)** AAO2-1 on-target site and **(B)** AAO_off-1 off-target candidate site using crRNA with guide sequences of different lengths. Mutation frequencies of selected calli (shown in red) were calculated from the ratio of sequenced clones with mutation. M, DNA molecular weight; –RE, without restriction enzyme; +RE, with restriction enzyme. Red arrowheads indicate the position of undigested PCR fragments. CAPS images of AAO2-1_Short and AAO2-1_Middle guides in AAO2-1 target site (A: upper and middle sides) and AAO2-1_Short and AAO2-1_Middle guides in AAO_off-1 target site (B: upper and middle sides) were grouping images from different parts of the same agarose gel electrophoresis image, respectively.

To investigate the effect of guide sequence length on off-target mutations, we focused on the *AAO* and *NCED* gene families (Tan et al., 2003; Hirano et al., 2008; Endo et al., 2016). We designed target sites in *AAO2* and *NECD1* genes as on-target (Table 3, Supplementary Table 4). The *AAO* gene family has three off-target candidate sites that have 1- or 2-nt mismatched sequence compared with the AAO2-1 guide sequence (AAO_off-1 to -3) (Table 3). We analyzed the mutation frequencies in these target sites by CAPS and sequence analysis (Figure 4). The mutation frequencies of the top two independent calli at on-target sites in *AAO2* were 67.7 and 83.8% in AAO2-1_Short, 23.3 and 58.1% in AAO2-1_Middle, and 22.5 and 43.3% in AAO2-1_Long, respectively (Figure 4A). On the other hand, at the off-target candidate sites (AAO_off-1 to -3), no undigested PCR fragments were detected for any guide length, meaning no mutation at these sites (Figure 4B, Supplementary Figure 9). *NCED2* and *NCED3* genes have 2-nt or 3-nt mismatched off-target candidate sites (NCED_off-1 and NCED_off-2) of NCED1-1 guide (Supplementary Table 4). Similar to the result of on- and off-target mutation analyses in the *AAO* gene family, mutations were clearly detected at the *NCED1* on-target site using NCED1-1_Short, _Middle, and _Long, and we could not detect any undigested fragment at the off-target candidate sites, even in NCED1-1_Short, by CAPS analysis (Supplementary Figure 10). Finally, we checked the genotypes of regenerated plants expressing AAO2-1_Short, AAO2-1_Long, NCED1-1_Short, and NCED1-1_Long, respectively, and no regenerated plants with off-target mutations were obtained (Supplementary Table 5). These results indicate that, while changing the length of the guide sequence could improve the mutation frequencies of on-target sites, it appears to have little effect on the accuracy of target sequence recognition of FnCas12a

**Table 3:** Target sequences of AAO2-1 on-target site and off-target candidate site.

**Target site**	**crRNA**	**PAM**	**On- or off-target sequence**
AAO2-1	AAO2-1_Short	TTG	GCAATGCTGTGTCATATG
	AAO2-1_Middle		GCAATGCTGTGTCATATGTTAATT
	AAO2-1_Long		GCAATGCTGTGTCATATGTTAATTCTGCAT
AAO_off-1	–	TTG	GCAATGCTGTTTCATATGTTAATTCTGCTT
AAO_off-2	–	TTG	GCAATGCTGTTTCATATGTTAATTCTGCTT
AAO_off-3	–	TTG	GCAATGCTGTCTCATATGTGAATTCTGCAT

*Red characters indicate mismatched nucleotide of off-target candidate sites*.

*Underlines at on- or off-target sequence indicate restriction enzyme sites for CAPS assay*.

## Discussion

In this study, we selected 10 target sites in five rice genes and showed that FnCas12a-mediated mutation efficiency could be improved by using different lengths of guide sequences. We detected mutations at eight target sites when using crRNA containing middle guide (24-nt). In four of the eight target sites (DL-1, DL-2, AAO2-1, and NCED1-1), short guide showed high mutation frequencies compared with middle guide (Figures 1, 4). On the other hand, using long guide improved mutation frequencies at the ALS-1 and ALS-2 sites (Supplementary Figure 3). These results suggest that, for efficient genome editing, an optimal length exists for each gene or target sequence. It would be useful if we could predict the best guide length *in silico*. Therefore, the secondary structure and GC contents of the crRNA were investigated for target sites whose mutation frequencies were improved by changing the length of the guide sequence (Supplementary Tables 6, 7). However, we were unable to find any relationship between these factors and mutation frequencies in our study. Previous studies have revealed that extension and modification of the 5′ and 3′ ends of the crRNA enhance the efficiency of AsCas12a-mediated genome editing in human cells (Moon et al., 2018; Park et al., 2018). These studies, together with our findings, emphasize the importance of designing crRNAs of appropriate length for each target sequence to further improve genome editing efficiency by FnCas12a. It has also been reported that the ability of Cas12a to self-process crRNA can be used to modify the crRNA expression vectors and improve the efficiency of multiple gene editing in plants (Tang et al., 2019; Xu et al., 2019). Combining these methods with our results may enable efficient multi-gene modification by FnCas12a.

Nucleotides 1–20 of the crRNA guide make an RNA–DNA heteroduplex with target DNA strands in AsCas12a and LbCas12a, and it has been suggested that Cas12a orthologs, including FnCas12a, recognize their target DNA region in a similar manner (Yamano et al., 2016, 2017). After forming the complex, FnCas12a introduces a DSB with a 5-nt 5′ overhang generated by cleaving after the 18th base on the non-targeted strand and after the 23rd base on the targeted strand from the PAM (Zetsche et al., 2015). However, Lei and colleagues reported that, when the guide sequence of crRNA was shorter than 20-nt, FnCas12a could cleave after the 14th base on the non-target strand from the PAM, generating longer 5′ overhangs (Lei et al., 2017). We showed that the frequency of large deletions was increased using the 18-nt short guide compared with the middle or long guide (Figure 2, Supplementary Figure 6), implying the importance of overhang length for deletion size. We also observed an increase in the frequency of deletions away from the PAM when using short guide (Figure 3, Supplementary Figure 8). It has been reported that SpCas9-RNA molecules remain tightly bound to the PAM-distal region after cleavage (Sternberg et al., 2014; Shibata et al., 2017). Since the DSB produced by FnCas12a was at the end of the target sequence, FnCas12a may continue to bind to the PAM side of the cleaved DNA, preventing DNA degradation at the PAM side.

It has been reported that shortened guide can reduce undesired mutagenesis at off-target sites in SpCas9-mediated genome editing (Fu et al., 2014). Furthermore, the off-target activity of Cas12a orthologs is relatively low compared with that of SpCas9 in human cells and plants (Endo et al., 2016; Kim et al., 2016, 2017; Kleinstiver et al., 2016). Consistent with these results, no mutations were introduced at off-target candidate sites with shortened guide in either rice calli or regenerated plants in our experiments (Figure 4, Supplementary Figure 10, Supplementary Table 5). The knowledge obtained in our study may help provide accurate genome editing with minimal off-target mutations. Further study is needed to clarify the relationship between off-target mutation and the length of guide sequence in FnCas12a-mediated genome editing.

It has been reported that 5′ sticky ends could increase the frequencies of targeted gene insertions and replacements via homologous recombination in the CRISPR/Cas9 paired nickase system (Bothmer et al., 2017). FnCas12a-mediated targeted gene insertions and replacements via homologous recombination have also been reported in rice (Begemann et al., 2017; Li et al., 2018). The FnCas12a-mediated genome editing platform has the potential to provide precise gene targeting with high frequencies. For this purpose, it is important to design effective crRNAs that can generate precise DSB at target sites. Our study results provide a basis for improved FnCas12a-mediated gene targeting efficiency through high efficiently and precise DSB induction.

## Data Availability Statement

The datasets presented in this study can be found in online repositories. The names of the repository/repositories and accession number(s) can be found below: DDBJ BioProject, PRJDB10598.

## Author Contributions

MM, ST, and ME designed the experiments. MM performed the experiments. KN and MM analyzed the results. KN and ME wrote the manuscript. All authors contributed to the article and approved the submitted version.

## Conflict of Interest

The authors declare that the research was conducted in the absence of any commercial or financial relationships that could be construed as a potential conflict of interest.

## References

[B1] Begemann M. Gray B. January E. Gordon G. He Y. Liu H.. (2017). Precise insertion and guided editing of higher plant genomes using Cpf1 CRISPR nucleases. Sci. Rep. 7:11606. 10.1038/s41598-017-11760-6 28912524 PMC5599503

[B2] Bothmer A. Phadke T. Barrera L. Margulies C. Lee C. Buquicchio F.. (2017). Characterization of the interplay between DNA repair and CRISPR/Cas9-induced DNA lesions at an endogenous locus. Nat. Commun. 8:13905. 10.1038/ncomms13905 28067217 PMC5227551

[B3] Dang Y. Jia G. Choi J. Ma H. Anaya E. Ye C.. (2015). Optimizing sgRNA structure to improve CRISPR-Cas9 knockout efficiency. Genome Biol. 16:280. 10.1186/s13059-015-0846-3 26671237 PMC4699467

[B4] Endo A. Masafumi M. Kaya H. Toki S. (2016). Efficient targeted mutagenesis of rice and tobacco genomes using Cpf1 from *Francisella novicida*. Sci. Rep. 6:38169. 10.1038/srep38169 27905529 PMC5131344

[B5] Endo M. Mikami M. Endo A. Kaya H. Itoh T. Nishimasu H.. (2019). Genome editing in plants by engineered CRISPR–Cas9 recognizing NG PAM. Nat. Plants 5, 14–17. 10.1038/s41477-018-0321-8 30531939

[B6] Fonfara I. Richter H. Bratovič M. Rhun A. Charpentier E. (2016). The CRISPR-associated DNA-cleaving enzyme Cpf1 also processes precursor CRISPR RNA. Nature 532, 517–521. 10.1038/nature17945 27096362

[B7] Fu Y. Sander J. Reyon D. Cascio V. Joung J. (2014). Improving CRISPR-Cas nuclease specificity using truncated guide RNAs. Nat. Biotechnol. 32, 279–284. 10.1038/nbt.2808 24463574 PMC3988262

[B8] Hendel A. Bak R. Clark J. Kennedy A. Ryan D. Roy S.. (2015). Chemically modified guide RNAs enhance CRISPR-Cas genome editing in human primary cells. Nat. Biotechnol. 33, 985–989. 10.1038/nbt.3290 26121415 PMC4729442

[B9] Hirano K. Aya K. Hobo T. Sakakibara H. Kojima M. Shim R.. (2008). Comprehensive transcriptome analysis of phytohormone biosynthesis and signaling genes in microspore/pollen and tapetum of rice. Plant Cell Physiol. 49, 1429–1450. 10.1093/pcp/pcn123 18718932 PMC2566925

[B10] Hu X. Meng X. Liu Q. Li J. Wang K. (2018). Increasing the efficiency of CRISPR-Cas9-VQR precise genome editing in rice. Plant Biotechnol. J. 16, 292–297. 10.1111/pbi.12771 28605576 PMC5785341

[B11] Jinek M. Chylinski K. Fonfara I. Hauer M. Doudna A. Charpentier E. (2012). A programmable dual-RNA-guided DNA endonuclease in adaptive bacterial immunity. Science 337, 816–821. 10.1126/science.1225829 22745249 PMC6286148

[B12] Kaya H. Mikami M. Endo A. Endo M. Toki S. (2016). Highly specific targeted mutagenesis in plants using *Staphylococcus aureus* Cas9. Sci. Rep. 6:26871. 10.1038/srep26871 27226350 PMC4881040

[B13] Kim H. Kim S. Ryu J. Kang B. Kim J. Kim S. (2017). CRISPR/Cpf1-mediated DNA-free plant genome editing. Nat. Commun. 8:14406. 10.1038/ncomms14406 28205546 PMC5316869

[B14] Kim Y. Cheong S. Lee J. Lee S. Lee M. Baek I.. (2016). Generation of knockout mice by Cpf1-mediated gene targeting. Nat. Biotechnol. 34, 808–810. 10.1038/nbt.3614 27272387

[B15] Kleinstiver B. Tsai S. Prew M. Nguyen N. Welch M. Lopez J.. (2016). Genome-wide specificities of CRISPR-Cas Cpf1 nucleases in human cells. Nat. Biotechnol. 34, 869–874. 10.1038/nbt.3620 27347757 PMC4980201

[B16] Lee K. Zhang Y. Kleinstiver B. Guo J. Aryee M. Miller J.. (2019). Activities and specificities of CRISPR/Cas9 and Cas12a nucleases for targeted mutagenesis in maize. Plant Biotechnol. J. 17, 362–372. 10.1111/pbi.12982 29972722 PMC6320322

[B17] Lei C. Li S. Liu J. Zheng X. Zhao G. Wang J. (2017). The CCTL (Cpf1-assisted cutting and Taq DNA ligase-assisted Ligation) method for efficient editing of large DNA constructs *in vitro*. Nucleic Acids Res. 45:9. 10.1093/nar/gkx018 28115632 PMC5436000

[B18] Li B. Zhao W. Luo X. Zhang X. Li C. Zeng C.. (2017). Engineering CRISPR-Cpf1 crRNAs and mRNAs to maximize genome editing efficiency. Nat. Biomed. Eng. 1:0066. 10.1038/s41551-017-0066 28840077 PMC5562407

[B19] Li J. Norville J. Aach J. McCormack M. Zhang D. Bush Z.. (2013). Multiplex and homologous recombination–mediated genome editing in *Arabidopsis* and *Nicotiana benthamiana* using guide RNA and Cas9. Nat. Biotechnol. 31, 688–691. 10.1038/nbt.2654 23929339 PMC4078740

[B20] Li S. Li J. Zhang J. Du W. Fu J. Sutar S.. (2018). Synthesis-dependent repair of Cpf1-induced double strand DNA breaks enables targeted gene replacement in rice. J. Exp. Bot. 69, 4715–4721. 10.1093/jxb/ery245 29955893 PMC6137971

[B21] Meng X. Hu X. Liu Q. Song X. Gao C. Li J.. (2018). Robust genome editing of CRISPR-Cas9 at NAG PAMs in rice. Sci. China Life Sci. 61, 122–125. 10.1007/s11427-017-9247-9 29285711

[B22] Mikami M. Toki S. Endo M. (2015). Comparison of CRISPR/Cas9 expression constructs for efficient targeted mutagenesis in rice. Plant Mol. Biol. 88, 561–572. 10.1007/s11103-015-0342-x 26188471 PMC4523696

[B23] Mikami M. Toki S. Endo M. (2017). In planta processing of the SpCas9-gRNA complex. Plant Cell Physiol. 58, 1857–1867. 10.1093/pcp/pcx154 29040704 PMC5921533

[B24] Moon S. Lee J. Kang J. Lee N. Ha D. Kim D.. (2018). Highly efficient genome editing by CRISPR-Cpf1 using CRISPR RNA with a uridinylate-rich 3′-overhang. Nat. Commun. 9, 3651. 10.1038/s41467-018-06129-w 30194297 PMC6128929

[B25] Nekrasov V. Staskawicz B. Weigel D. Jones J. Kamoun S. (2013). Targeted mutagenesis in the model plant *Nicotiana benthamiana* using Cas9 RNA-guided endonuclease. Nat. Biotechnol. 31, 691–693. 10.1038/nbt.2655 23929340

[B26] Park H. Liu H. Wu J. Chong A. Mackley V. Fellmann C.. (2018). Extension of the crRNA enhances Cpf1 gene editing in vitro and in vivo. Nat. Commun. 9:3313. 10.1038/s41467-018-05641-3 30120228 PMC6098076

[B27] Ryan D. Taussig D. Steinfeld I. Phadnis S. Lunstad B. Singh M.. (2018). Improving CRISPR-Cas specificity with chemical modifications in single-guide RNAs. Nucleic Acids Res. 46, 792–803. 10.1093/nar/gkx1199 29216382 PMC5778453

[B28] Shan Q. Wang Y. Li J. Zhang Y. Chen K. Liang Z.. (2013). Targeted genome modification of crop plants using a CRISPR-Cas system. Nat. Biotechnol. 31, 686–688. 10.1038/nbt.2650 23929338

[B29] Shibata M. Nishimasu H. Kodera N. Hirano S. Ando T. Uchihashi T.. (2017). Real-space and real-time dynamics of CRISPR-Cas9 visualized by high-speed atomic force microscopy. Nat. Commun. 8:1430. 10.1038/s41467-017-01466-8 29127285 PMC5681550

[B30] Steinert J. Schiml S. Fauser F. Puchta H. (2015). Highly efficient heritable plant genome engineering using Cas9 orthologues from *Streptococcus thermophilus* and *Staphylococcus aureus*. Plant J. 84, 1295–1305. 10.1111/tpj.13078 26576927

[B31] Sternberg S. Redding S. Jinek M. Greene E. Doudna J. (2014). DNA interrogation by the CRISPR RNA-guided endonuclease Cas9. Nature 507, 62–67. 10.1038/nature13011 24476820 PMC4106473

[B32] Tan B. Joseph L. Deng W. Liu L. Li Q. Cline K.. (2003). Molecular characterization of the *Arabidopsis* 9-*cis* epoxycarotenoid dioxygenase gene family. Plant J. 35, 44–56. 10.1046/j.1365-313X.2003.01786.x 12834401

[B33] Tang X. Lowder L. Zhang T. Malzahn A. Zheng X. Voytas D.. (2017). A CRISPR-Cpf1 system for efficient genome editing and transcriptional repression in plants. Nat. Plants 3:17018. 10.1038/nplants.2017.18 28211909

[B34] Tang X. Ren Q. Yang L. Bao Y. Zhong Z. He Y.. (2019). Single transcript unit CRISPR 2.0 systems for robust Cas9 and Cas12a mediated plant genome editing. Plant Biotechnol. J. 17, 1431–1445. 10.1111/pbi.13068 30582653 PMC6576101

[B35] Toki S. (1997). Rapid and efficient *Agrobacterium*-mediated transformation in rice. Plant Mol. Biol. Rep. 15, 16–21. 10.1007/BF02772109

[B36] Toki S. Hara N. Ono K. Onodera H. Tagiri A. Oka S.. (2006). Early infection of scutellum tissue with *Agrobacterium* allows high-speed transformation of rice. Plant J. 47, 969–976. 10.1111/j.1365-313X.2006.02836.x 16961734

[B37] Wang M. Mao Y. Lu Y. Tao X. Zhu J. (2017). Multiplex gene editing in rice using the CRISPR-Cpf1 system. Mol. Plant 10, 1011–1013. 10.1016/j.molp.2017.03.001 28315752

[B38] Xu R. Qin R. Li H. Li D. Li L. Wei P.. (2017). Generation of targeted mutant rice using a CRISPR-Cpf1 system. Plant Biotechnol. J. 15, 713–717. 10.1111/pbi.12669 27875019 PMC5425385

[B39] Xu R. Qin R. Li H. Li J. Yang J. Wei P. (2019). Enhanced genome editing in rice using single transcript unit CRISPR-*Lb*Cpf1 systems. Plant Biotechnol. J. 17, 553–555. 10.1111/pbi.13028 30367555 PMC6381782

[B40] Yamano T. Nishimasu H. Zetsche B. Hirano H. Slaymaker I. Li Y.. (2016). Crystal *s*tructure of Cpf1 in complex with guide RNA and target DNA. Cell 165, 949–962. 10.1016/j.cell.2016.04.003 27114038 PMC4899970

[B41] Yamano T. Zetsche B. Ishitani R. Zhang F. Nishimasu H. Nureki O. (2017). Structural basis for the Canonical and non-Canonical PAM Recognition by CRISPR-Cpf1. Mol. Cell 67, 633–645. 10.1016/j.molcel.2017.06.035 28781234 PMC5957536

[B42] Zetsche B. Gootenberg J. Abudayyeh O. Slaymaker I. Makarova K. Essletzbichler P.. (2015). Cpf1 is a single RNA-guided endonuclease of a class 2 CRISPR-Cas system. Cell 163, 759–771. 10.1016/j.cell.2015.09.038 26422227 PMC4638220

