# Supplementary material for: Enhanced FnCas12a-Mediated Targeted Mutagenesis Using crRNA With Altered Target Length in Rice
Source: Front Genome Ed. 2020 Dec 14;2:608563. doi: 10.3389/fgeed.2020.608563 (PMC8525410; doi:10.3389/fgeed.2020.608563)
Supplement: Supplementary file 1 [file Data_Sheet_1.PDF]

Supplementary Material

(A)

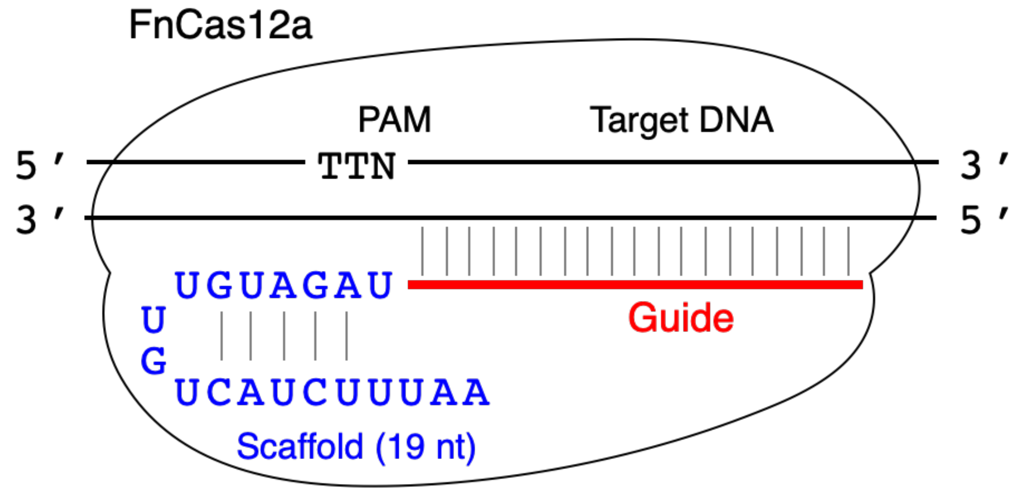

(B)

Guide	length (nt)	Ratio of guide in crRNA (%)
Short	18	48.6
Middle	24	55.8
Long	30	61.2
Very long	45	70.3

Figure S1. crRNA sequence in FnCas12a-mediated genome editing.
(A) Target DNA recognition by FnCas12a. Blue characters indicate the 19-nt scaffold sequence in crRNA. The red bar indicates the crRNA guide sequence, which is complementary to the sequence of the target DNA.
(B) Guide type and sequence length using in this study. Ratio of guide in crRNA means guide sequence length per total crRNA length.

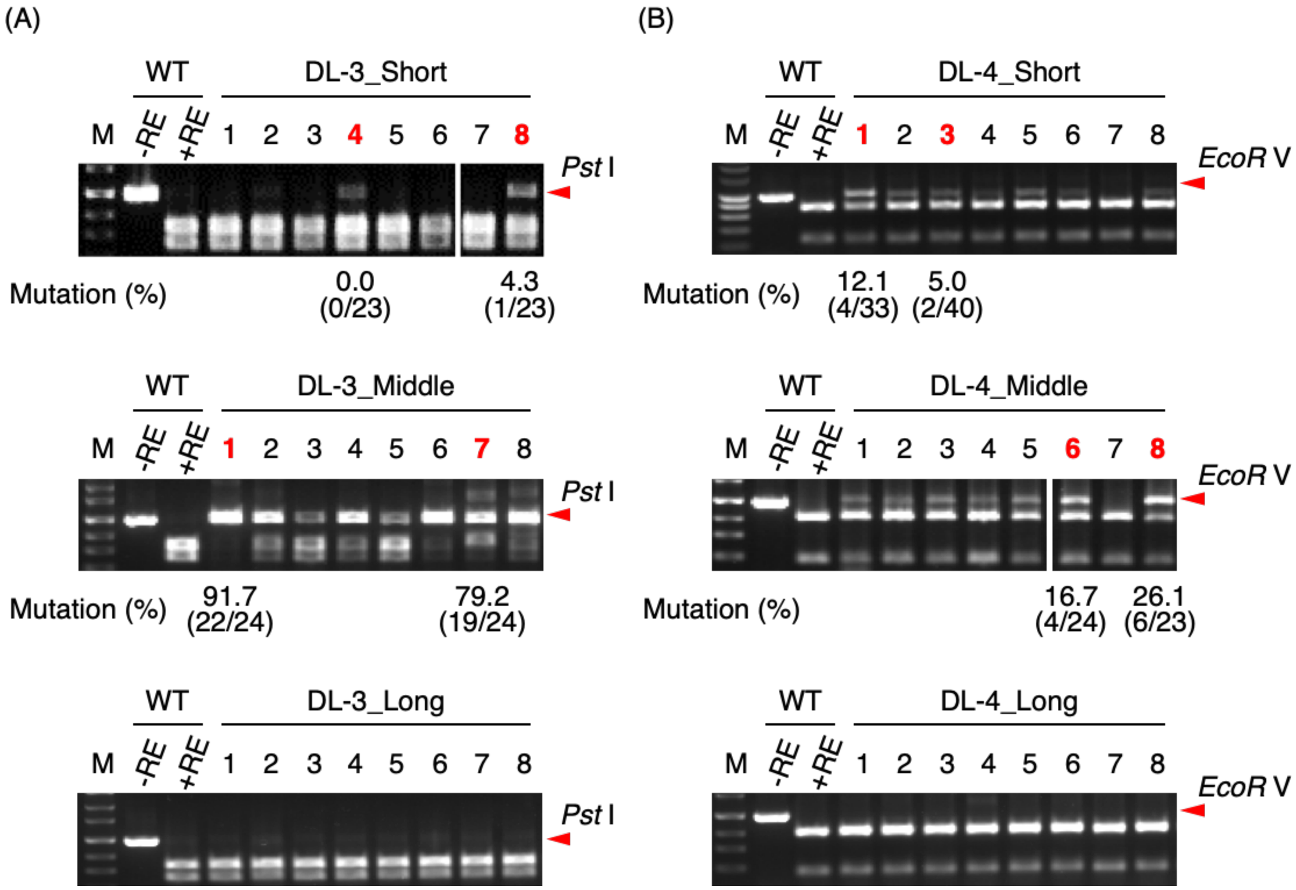

Figure S2. CAPS analysis and mutation frequency using crRNA with guide sequences of different lengths at DL-3 (A) and DL-4 (B) target sites.

Mutation frequencies of selected calli (shown in red) were calculated from the ratio of sequenced clones with mutation. M: DNA molecular weight. –RE: without restriction enzyme. +RE: with restriction enzyme. Red arrowheads indicate the position of undigested PCR fragments. The CAPS image of DL-3_Short (A: upper side) and DL-4_Middle (B: middle side) were assembled from different parts of the same agarose gel electrophoresis image.

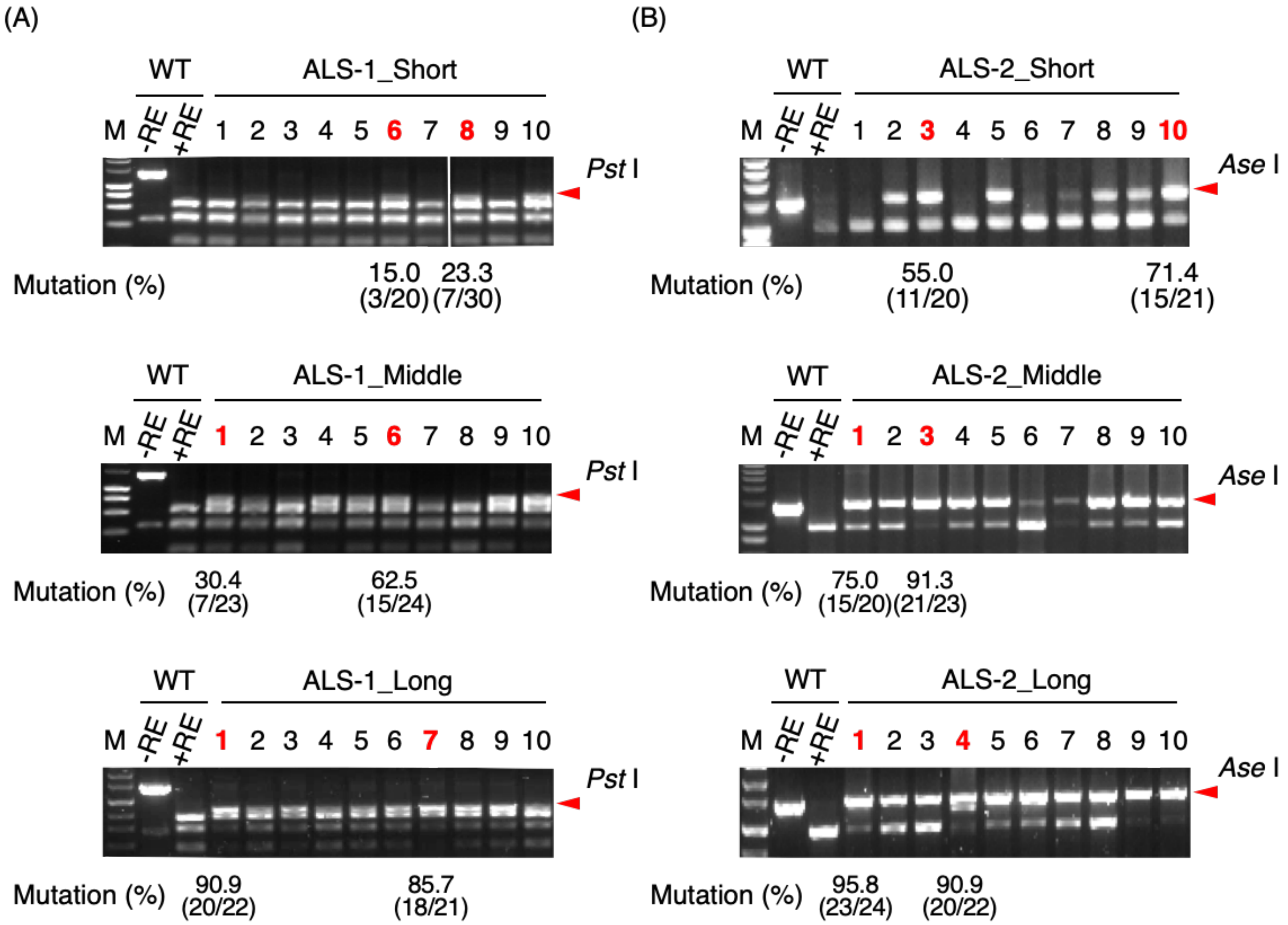

Figure S3. CAPS analysis and mutation frequency using crRNA with guide sequences of different lengths at ALS-1 (A) and ALS-2 (B) target sites.

Mutation frequencies of selected calli (shown in red) were calculated from the ratio of sequenced clones with mutation. M: DNA molecular weight. –RE: without restriction enzyme. +RE: with restriction enzyme. Red arrowheads indicate the position of undigested PCR fragments. The CAPS image of ALS-1_Short (A: upper side) was assembled from different parts of the same agarose gel electrophoresis image.

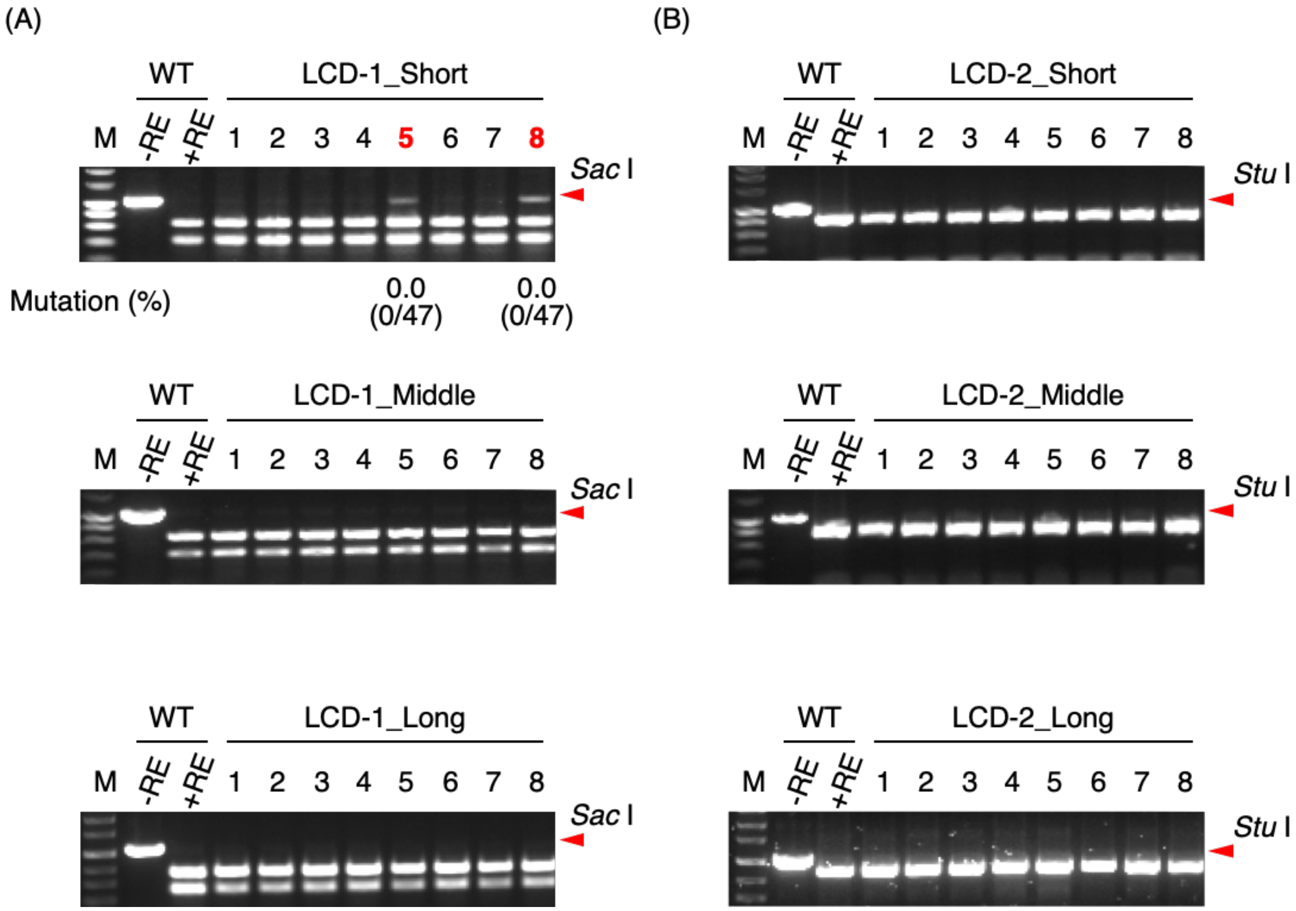

Figure S4. CAPS analysis and mutation frequency using crRNA with guide sequences of different lengths at LCD-1 (A) and LCD-2 (B) target sites.

Mutation frequencies of selected calli (shown in red) were calculated from the ratio of sequenced clones with mutation. M: DNA molecular weight. –RE: without restriction enzyme. +RE: with restriction enzyme. Red arrowheads indicate the position of undigested PCR fragments.

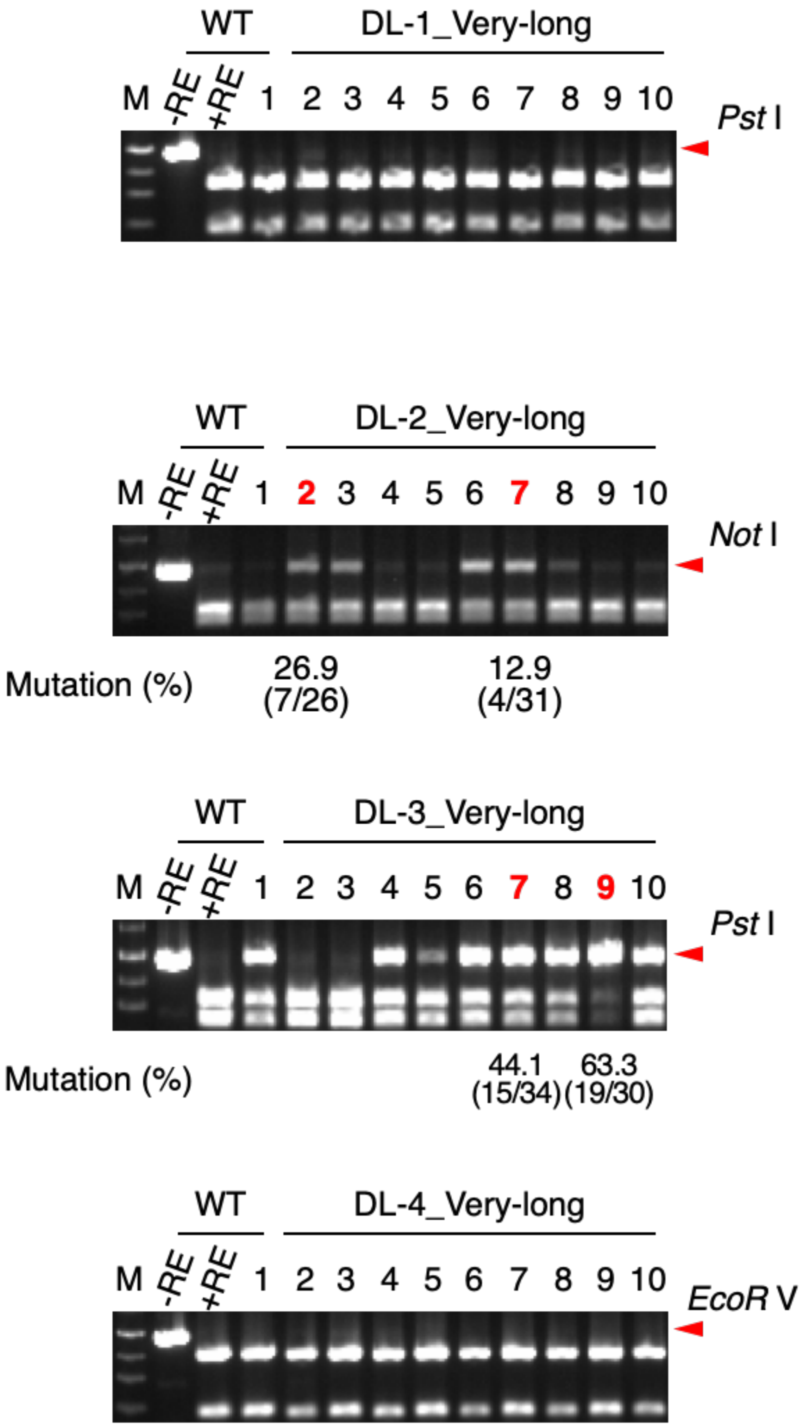

Figure S5. CAPS analysis and mutation frequency using very long guide at DL-1 to -4 target sites. Mutation frequencies of selected calli (shown in red) were calculated from the ratio of sequenced clones with mutation. M: DNA molecular weight. –RE: without restriction enzyme. +RE: with restriction enzyme. Red arrowheads indicate the position of undigested PCR fragments.

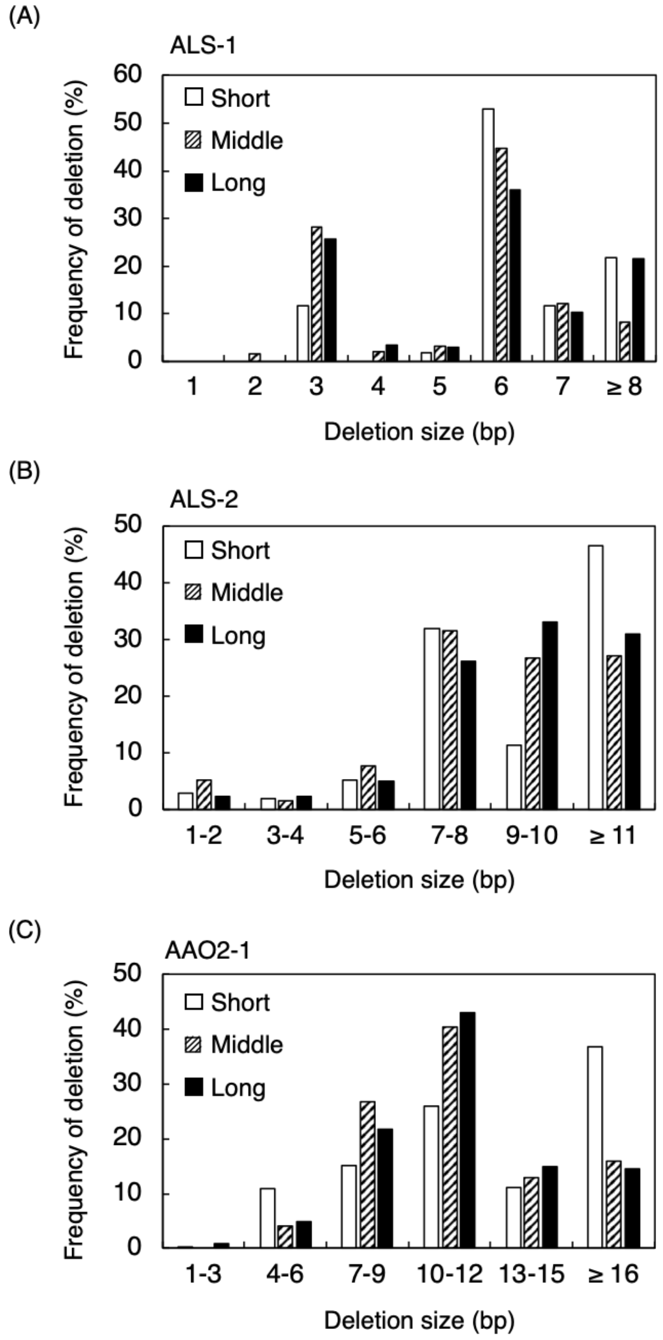

Figure S6. Comparison of deletion sizes using crRNA with guide sequences of different lengths at (A) ALS-1, (B) ALS-2, and (C) AAO2-1 target sites.
Deletion size and mutation number were detected by targeted amplicon sequencing. Frequency of deletion means the percentage of deletions within the range of each deletion sizes per total number of deletion mutations.

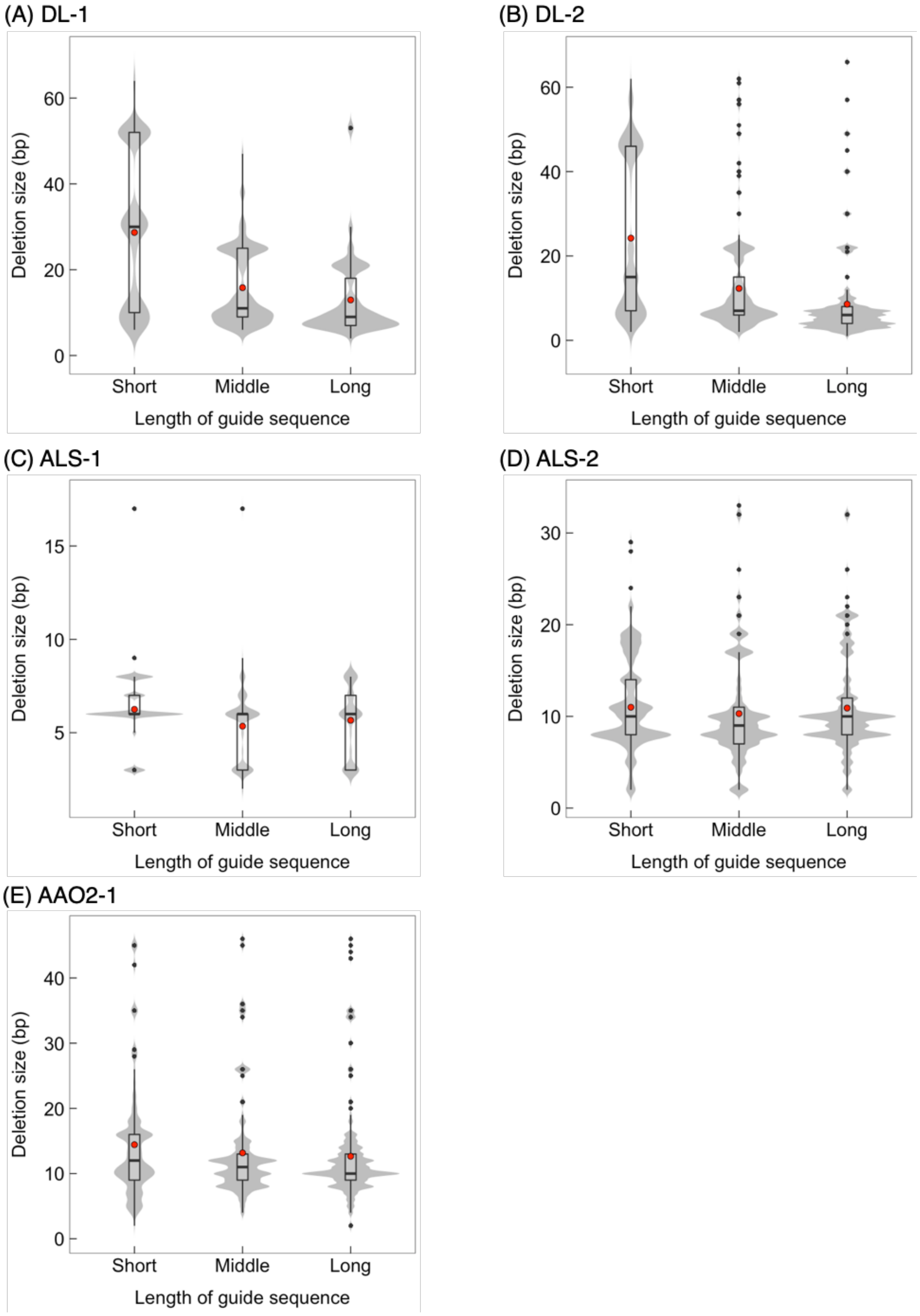

Figure S7. Summary of the distribution of deletion size at (A) DL-1, (B) DL-2, (C) ALS-1, (D) ALS-2 and (E) AAO2-1 target sites.

The distribution of deletion size is shown on a violin plot (gray) and box plot. The middle line of box plot represents the median and outliers are shown as individual black dots. The red dot represents mean (average deletion size).

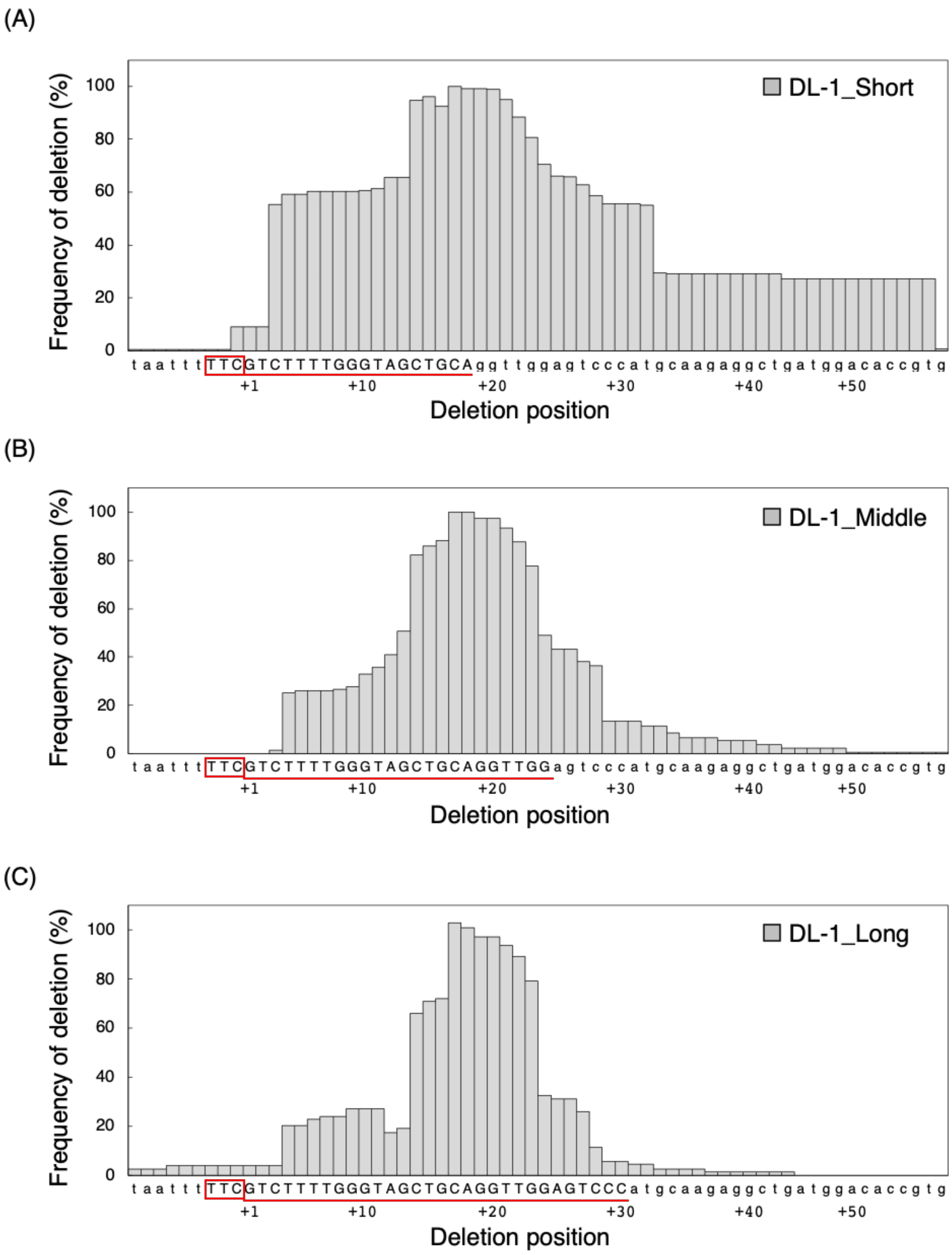

Figure S8. Comparison of deletion position according to the (A) DL1-Short (B) DL-1_Middle (C) DL-1_Long guide at DL-1 target sites.

Deletion size and mutation number were detected by targeted amplicon sequencing. Frequency of deletion means the percentage of deletions in each position among all deletion mutations. The target sequence of each guide is shown in red, underlined and in capital letters. The PAM sequence upstream of the target sequence is boxed in red.

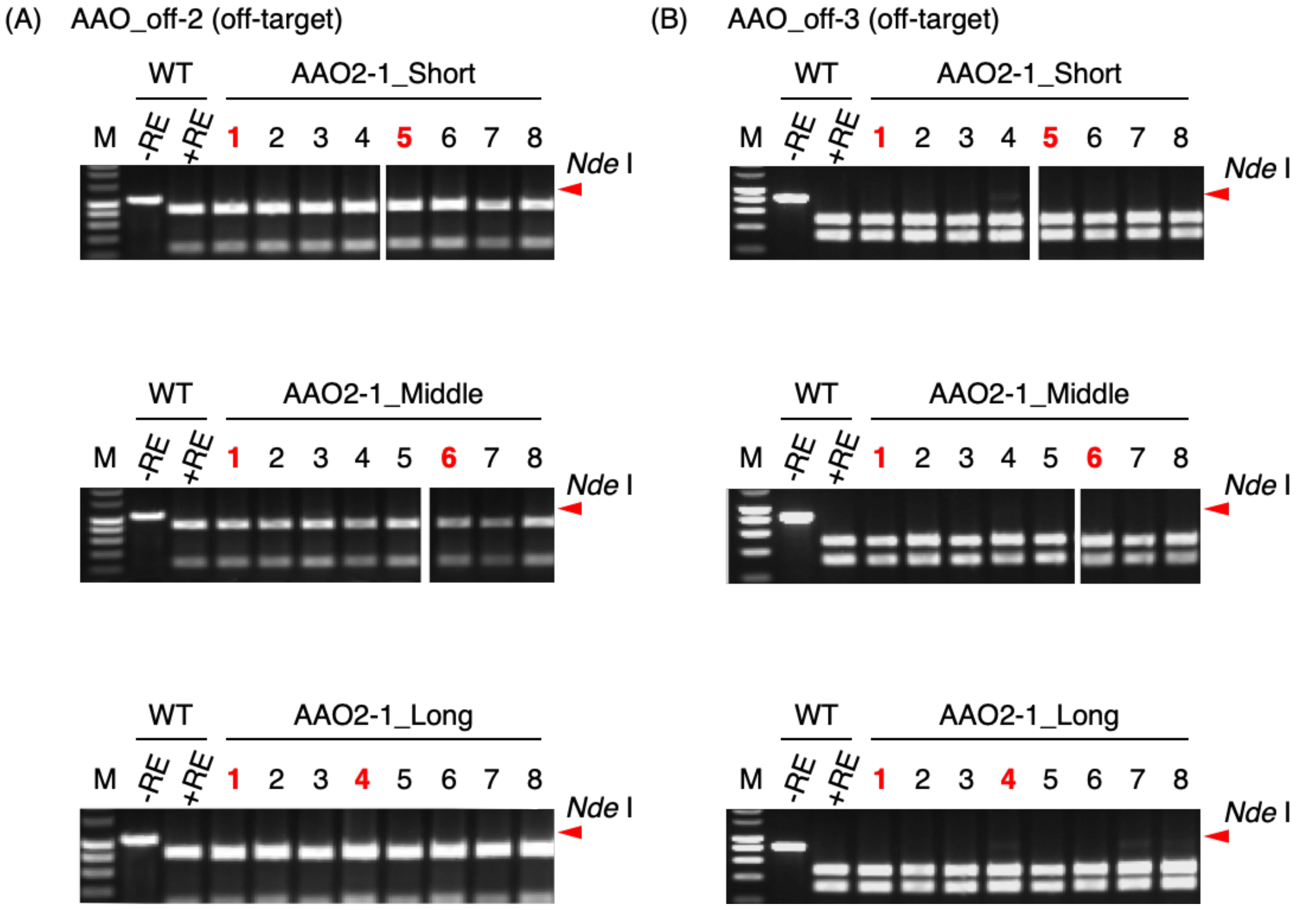

Figure S9. Off-target mutation analysis using CAPS assay at (A) AAO_off-2 and (B) AAO_off-3 off-target candidate sites using crRNA with guide sequences of different lengths.
Transgenic lines shown in red number indicate the selected calli which were investigated mutation frequencies at AAO2-1 on-target sites . M: DNA molecular weight. –RE: without restriction enzyme. +RE: with restriction enzyme. Red arrowheads indicate the position of undigested PCR fragments. CAPS images of AAO2-1_Short and AAO2-1_Middle guide in AAO_off-2 target site (A: upper and middle side) and AAO2-1_Short and AAO2-1_Middle guide in AAO_off-3 target site (B: upper and middle side) were grouping images from different parts of the same agarose gel electrophoresis image, respectively.

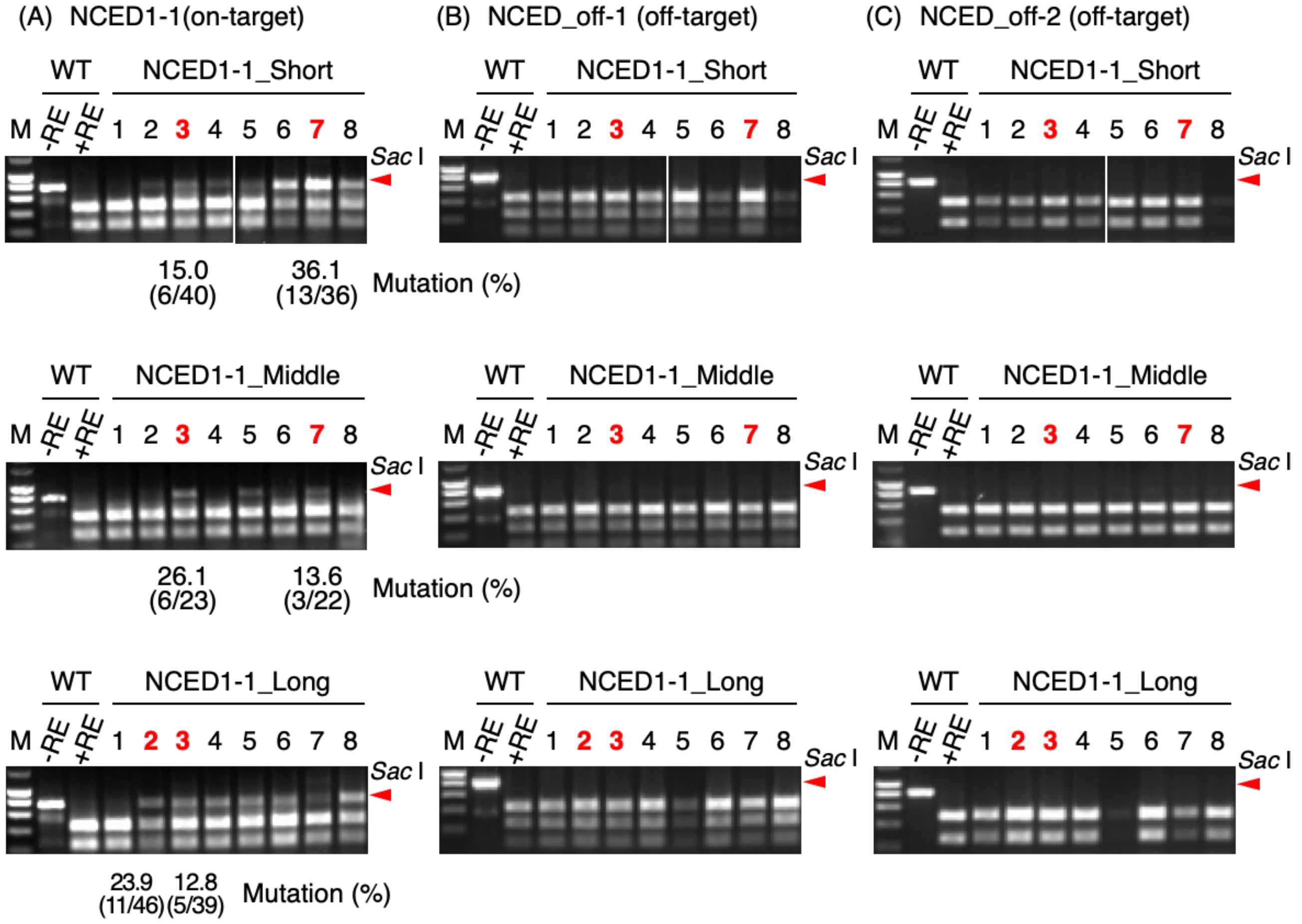

Figure S10. Off-target mutation analysis using CAPS assay at (A) NCED1-1 on-target site and (B) NCED_off-1 and (C) NCED_off-2 off-target candidate sites using crRNA with guide sequences of different lengths.

Mutation frequencies of selected calli (shown in red) were calculated from the ratio of sequenced clones with mutation. M: DNA molecular weight. –RE: without restriction enzyme. +RE: with restriction enzyme. Red arrowheads indicate the position of undigested PCR fragments. CAPS images of NCED1-1_Short guide in NCED1-1 target site (A: upper side) and NCED1-1_Short in NCED_off-1 target site (B: upper side) and NCED1-1_Short in NCED_off-2 target site (C: upper side) were assembled from different parts of the same agarose gel electrophoresis image, respectively.

Table S1. Oligonucleotides and Primers used in this study (continued on next page).

Oligonucleotide or Primer	Used for	Sequence
DL-1_Short-F	Cloning crRNA	AGATGTCTTTTGGGTAGCTGCA
DL-1_Short-R	Cloning crRNA	AAAATGCAGCTACCCAAAAGAC
DL-1_Middle-F	Cloning crRNA	AGATGTCTTTTGGGTAGCTGCAGGTTGG
DL-1_Middle-R	Cloning crRNA	AAAACCAACCTGCAGCTACCCAAAAGAC
DL-1_Long-F	Cloning crRNA	AGATGTCTTTTGGGTAGCTGCAGGTTGGAGTCCC
DL-1_Long-R	Cloning crRNA	AAAAGGGACTCCAACCTGCAGCTACCCAAAAGAC
DL-1_Very-long-F	Cloning crRNA	AGATGTCTTTTGGGTAGCTGCAGGTTGGAGTCCCATGCAAGAGGCTGAT
DL-1_Very-long-R	Cloning crRNA	AAAAATCAGCCTCTTGCATGGGACTCCAACCTGCAGCTACCCAAAAGAC
DL-2_Short-F	Cloning crRNA	AGATGGGAGAGCGGCTGCACCA
DL-2_Short-R	Cloning crRNA	AAAATGGTGCAGCCGCTCTCCC
DL-2_Middle-F	Cloning crRNA	AGATGGGAGAGCGGCTGCACCATCGGCG
DL-2_Middle-R	Cloning crRNA	AAAACGCCGATGGTGCAGCCGCTCTCCC
DL-2_Long-F	Cloning crRNA	AGATGGGAGAGCGGCTGCACCATCGGCGGCCGCG
DL-2_Long-R	Cloning crRNA	AAAACGCGGCCGCCGATGGTGCAGCCGCTCTCCC
DL-2_Very-long-F	Cloning crRNA	AGATGGGAGAGCGGCTGCACCATCGGCGGCCGCGGGCTGAGGAAGGAGA
DL-2_Very-long-R	Cloning crRNA	AAAATCTCCTTCCTCAGCCCGCGGCCGCCGATGGTGCAGCCGCTCTCCC
DL-3_Short-F	Cloning crRNA	AGATGGGACCTTGCACTGACTG
DL-3_Short-R	Cloning crRNA	AAAACAGTCAGTGCAAGGTCCC
DL-3_Middle-F	Cloning crRNA	AGATGGGACCTTGCACTGACTGCAGGAG
DL-3_Middle-R	Cloning crRNA	AAAACTCCTGCAGTCAGTGCAAGGTCCC
DL-3_Long-F	Cloning crRNA	AGATGGGACCTTGCACTGACTGCAGGAGGAACCA
DL-3_Long-R	Cloning crRNA	AAAATGGTTCCTCCTGCAGTCAGTGCAAGGTCCC
DL-3_Very-long-F	Cloning crRNA	AGATGGGACCTTGCACTGACTGCAGGAGGAACCAGCCGCTGCCGCTGGT
DL-3_Very-long-R	Cloning crRNA	AAAAACCAGCGGCAGCGGCTGGTTCCTCCTGCAGTCAGTGCAAGGTCCC
DL-4_Short-F	Cloning crRNA	AGATATTAGAAAGATATTGATA
DL-4_Short-R	Cloning crRNA	AAAATATCAATATCTTTCTAAT
DL-4_Middle-F	Cloning crRNA	AGATATTAGAAAGATATTGATATCTACT
DL-4_Middle-R	Cloning crRNA	AAAAAGTAGATATCAATATCTTTCTAAT
DL-4_Long-F	Cloning crRNA	AGATATTAGAAAGATATTGATATCTACTCTAGCT
DL-4_Long-R	Cloning crRNA	AAAAAGCTAGAGTAGATATCAATATCTTTCTAAT
DL-4_Very-long-F	Cloning crRNA	AGATATTAGAAAGATATTGATATCTACTCTAGCTGGTATATATGCACGT
DL-4_Very-long-R	Cloning crRNA	AAAAACGTGCATATATACCAGCTAGAGTAGATATCAATATCTTTCTAAT
ALS-1_Short-F	Cloning crRNA	AGATGCCACAGAAGCACCAGCT
ALS-1_Short-R	Cloning crRNA	AAAAAGCTGGTGCTTCTGTGGC
ALS-1_Middle-F	Cloning crRNA	AGATGCCACAGAAGCACCAGCTGCAGCA
ALS-1_Middle-R	Cloning crRNA	AAAATGCTGCAGCTGGTGCTTCTGTGGC
ALS-1_Long-F	Cloning crRNA	AGATGCCACAGAAGCACCAGCTGCAGCAGGCAGC
ALS-1_Long-R	Cloning crRNA	AAAAGCTGCCTGCTGCAGCTGGTGCTTCTGTGGC
ALS-2_Short-F	Cloning crRNA	AGATCCAACATACAGATTATAG
ALS-2_Short-R	Cloning crRNA	AAAACTATAATCTGTATGTTGG
ALS-2_Middle-F	Cloning crRNA	AGATCCAACATACAGATTATAGATTAAT
ALS-2_Middle-R	Cloning crRNA	AAAAATTAATCTATAATCTGTATGTTGG
ALS-2_Long-F	Cloning crRNA	AGATCCAACATACAGATTATAGATTAATACACAG
ALS-2_Long-R	Cloning crRNA	AAAACTGTGTATTAATCTATAATCTGTATGTTGG
AAO2-1_Short-F	Cloning crRNA	AGATGCAATGCTGTGTCATATG
AAO2-1_Short-R	Cloning crRNA	AAAACATATGACACAGCATTGC
AAO2-1_Middle-F	Cloning crRNA	AGATGCAATGCTGTGTCATATGTTAATT
AAO2-1_Middle-R	Cloning crRNA	AAAAAATTAACATATGACACAGCATTGC
AAO2-1_Long-F	Cloning crRNA	AGATGCAATGCTGTGTCATATGTTAATTCTGCAT
AAO2-1_Long-R	Cloning crRNA	AAAAATGCAGAATTAACATATGACACAGCATTGC
NCED1-1_Short-F	Cloning crRNA	AGATCCCAAGGCCATTGGGGAG
NCED1-1_Short-R	Cloning crRNA	AAAACTCCCCAATGGCCTTGGG
NCED1-1_Middle-F	Cloning crRNA	AGATCCCAAGGCCATTGGGGAGCTCCAT
NCED1-1_Middle-R	Cloning crRNA	AAAAATGGAGCTCCCCAATGGCCTTGGG
NCED1-1_Long-F	Cloning crRNA	AGATCCCAAGGCCATTGGGGAGCTCCATGGCCAC
NCED1-1_Long-R	Cloning crRNA	AAAAGTGGCCATGGAGCTCCCCAATGGCCTTGGG

Table S1. Oligonucleotides and Primers used in this study.

Oligonucleotide or Primer	Used for	Sequence
LCD-1_Short-F	Cloning crRNA	AGATGAAACAGGTGGAAAAGAG
LCD-1_Short-R	Cloning crRNA	AAAACTCTTTTCCACCTGTTTC
LCD-1_Middle-F	Cloning crRNA	AGATGAAACAGGTGGAAAAGAGCTCTGC
LCD-1_Middle-R	Cloning crRNA	AAAAGCAGAGCTCTTTTCCACCTGTTTC
LCD-1_Long-F	Cloning crRNA	AGATGAAACAGGTGGAAAAGAGCTCTGCTTTGAC
LCD-1_Long-R	Cloning crRNA	AAAAGTCAAAGCAGAGCTCTTTTCCACCTGTTTC
LCD-2_Short-F	Cloning crRNA	AGATTTCTTTACACCAAGTCAT
LCD-2_Short-R	Cloning crRNA	AAAAATGACTTGGTGTAAAGAA
LCD-2_Middle-F	Cloning crRNA	AGATTTCTTTACACCAAGTCATGAGGCC
LCD-2_Middle-R	Cloning crRNA	AAAAGGCCTCATGACTTGGTGTAAAGAA
LCD-2_Long-F	Cloning crRNA	AGATTTCTTTACACCAAGTCATGAGGCCTTGGAC
LCD-2_Long-R	Cloning crRNA	AAAAGTCCAAGGCCTCATGACTTGGTGTAAAGAA
DL-1and2-F	CAPS assay	CAGTGTCATGTTCCATCTTTCGCTTCCATT
DL-1and2-R	CAPS assay	ATGGGCAAGAGAGAAATCTTTTGCAATCCA
DL-3and4-F	CAPS assay	TGCAAAAGATTTCTCTCTTGCCCATCTGTG
DL-3and4-R	CAPS assay	TTTCTCACCTCATGAAGCGGTTGTAAGCAG
ALS-1_and_2-F	CAPS assay	AATTATGCCGTGGATAAGGCTGACCTGTTG
ALS-1_and_2-R	CAPS assay	ACCCAATAAGATCGACCGAAGAGAGGGAAA
AAO2-1-F	CAPS assay	ACATCATCATGGCGCAGAGGCTGTCTTTCC
AAO2-1-R	CAPS assay	TCTAAGATGCTCTAACATGGGTAGCACGAA
AAO_off-1_and_off-2 -F	CAPS assay	GGCACACAAATACCCTTTTCGCTCAGACAT
AAO_off-1-R	CAPS assay	TATCCTTGGCAGAAACAACTGTAAGGATCTTCTTT
AAO_off-2-R	CAPS assay	ACCACAACACCGAGAACTTGTCCAGCAAAC
AAO_off-3-F	CAPS assay	CAAAGCCATCGAGATACTACGGTCAGATGG
AAO_off-3-R	CAPS assay	CGACTTTCTTGATTGGTTTTCCGACTGGTT
NCED1-1-F	CAPS assay	GTCGGGGAAGAGTGCCATTGGCGTCCCGAA
NCED1-1-R	CAPS assay	CTTCTTGATCACGTCGTAGCTGAGCGCGTG
NCED_off-1-F	CAPS assay	CGGCGAGGAGGTGTGGCAAGAAGAAGGATG
NCED_off-1-R	CAPS assay	GGCTGCTCAAGCTCGATCTCGACGTCGGGT
NCED_off-2-F	CAPS assay	GCAACCGTGCCAACCAATGGCAAGATCAAG
NCED_off-2-R	CAPS assay	CCGTCGGCGGTGAAGTAGAAGTACTTGAGG
LCD-1_and_2-F	CAPS assay	ACTAGTGCCAGGGCAGCAATAATGGATTTG
LCD-1_and_2-R	CAPS assay	CACAAACGGCCAATGAAAATCATCCCTACT
DL-1_NGS-F	Amplicon sequencing	ACACTCTTTCCCTACACGACGCTCTTCCGATCTGTTTTGCATATCCCACTTTATTTGCTTGCTCCATC
DL-1_NGS-R	Amplicon sequencing	GTGACTGGAGTTCAGACGTGTGCTCTTCCGATCTTTAGCTAGAGGAGAGGACTGACCTGAAACG
DL-2_NGS-F	Amplicon sequencing	ACACTCTTTCCCTACACGACGCTCTTCCGATCTACGAAGAAAGTATTGGCTTAATTGCATGAT
DL-2_NGS-R	Amplicon sequencing	GTGACTGGAGTTCAGACGTGTGCTCTTCCGATCTAAACAGCAACACGAGAACTGAAGAAGATCA
ALS-1_NGS-F	Amplicon sequencing	ACACTCTTTCCCTACACGACGCTCTTCCGATCTGAAAGGTGAGGCAATCATCGCTACTGGTGT
ALS-1_NGS-R	Amplicon sequencing	GTGACTGGAGTTCAGACGTGTGCTCTTCCGATCTACCATACCCAAATGTTGGTTGTTCAACACC
ALS-2_NGS-F	Amplicon sequencing	ACACTCTTTCCCTACACGACGCTCTTCCGATCTTGCCGCCATCAAGAAGATGCTCGAGACTCC
ALS-2_NGS-R	Amplicon sequencing	GTGACTGGAGTTCAGACGTGTGCTCTTCCGATCTCATGCCAAGCACATCAAACAAGTAAAACAG
AAO2-1_NGS-F	Amplicon sequencing	ACACTCTTTCCCTACACGACGCTCTTCCGATCTGCAGCCTCCATGTGATTCCAGGACGTTGTT
AAO2-1_NGS-R	Amplicon sequencing	GTGACTGGAGTTCAGACGTGTGCTCTTCCGATCTTGGTGAAACAACACCTTTAAGAAGCCGAAC

Table S2. Number of samples for CAPS analysis.

Target site	crRNA	Sample number for CAPS analysis	Number of samples with undigested PCR fragment	Sequenced line
DL-1	Short	24	24	#2, #7
	Middle	24	8	#5, #8
	Long	24	15	#5, #10
	Very long	24	0	-
DL-2	Short	24	23	#4, #6
	Middle	24	20	#3, #5
	Long	24	17	#1, #4
	Very long	24	14	#2, #7
DL-3	Short	24	5	#4, #8
	Middle	24	24	#1, #7
	Long	24	0	-
	Very long	24	18	#7, #9
DL-4	Short	21	13	#1, #3
	Middle	24	18	#6. #8
	Long	24	0	-
	Very long	24	0	-
ALS1-1	Short	21	10	#6, #8
	Middle	18	12	#1, #6
	Long	24	21	#1, #7
ALS1-2	Short	24	16	#3, #10
	Middle	18	18	#1, #3, #10
	Long	24	23	#1, #4
LCD-1	Short	21	4	#5, #8
	Middle	21	0	-
	Long	21	0	-
LCD-2	Short	21	0	-
	Middle	21	0	-
	Long	21	0	-
AAO2-1	Short	23	19	#1, #5
	Middle	18	15	#1, #6
	Long	23	21	#1, #4
NCED1-1	Short	23	12	#3, #7
	Middle	18	5	#3, #7
	Long	23	15	#2, #3

Table S3. Target sites and sequences.

Target gene	crRNA	PAM	Sequence	Length (nt)
DL	DL-1_Very-long	TTC	GTCTTTTGGGTAGCTGCAGGTTGGAGTCCCATGCAAGAGGCTGAT	45
	DL-2_Very-long	TTG	GGGAGAGCGGCTGCACCATCGGCGGCCGCGGGCTGAGGAAGGAGA	45
	DL-3_Short	TTC	GGGACCTTGCACTGACTG	18
	DL-3_Middle		GGGACCTTGCACTGACTGCAGGAG	24
	DL-3_Long		GGGACCTTGCACTGACTGCAGGAGGAACCA	30
	DL-3_Very-long		GGGACCTTGCACTGACTGCAGGAGGAACCAGCCGCTGCCGCTGGT	45
	DL-4_Short	TTA	ATTAGAAAGATATTGATA	18
	DL-4_Middle		ATTAGAAAGATATTGATATCTACT	24
	DL-4_Long		ATTAGAAAGATATTGATATCTACTCTAGCT	30
	DL-4_Very-long		ATTAGAAAGATATTGATATCTACTCTAGCTGGTATATATGCACGT	45
ALS	ALS-1_Short	TTG	GCCACAGAAGCACCAGCT	18
	ALS-1_Middle		GCCACAGAAGCACCAGCTGCAGCA	24
	ALS-1_Long		GCCACAGAAGCACCAGCTGCAGCAGGCAGC	30
	ALS-2_Short	TTC	CCAACATACAGATTATAG	18
	ALS-2_Middle		CCAACATACAGATTATAGATTAAT	24
	ALS-2_Long		CCAACATACAGATTATAGATTAATACACAG	30
LCD	LCD-1_Short	TTC	GAAACAGGTGGAAAAGAG	18
	LCD-1_Middle		GAAACAGGTGGAAAAGAGCTCTGC	24
	LCD-1_Long		GAAACAGGTGGAAAAGAGCTCTGCTTTGAC	30
	LCD-2_Short	TTG	TTCTTTACACCAAGTCAT	18
	LCD-2_Middle		TTCTTTACACCAAGTCATGAGGCC	24
	LCD-2_Long		TTCTTTACACCAAGTCATGAGGCCTTGGAC	30
NCED1	NCED1-1_Short	TTC	CCCAAGGCCATTGGGGAG	18
	NCED1-1_Middle		CCCAAGGCCATTGGGGAGCTCCAT	24
	NCED1-1_Long		CCCAAGGCCATTGGGGAGCTCCATGGCCAC	30

Underlines at on- or off-target sequences indicate the restriction enzyme sites for CAPS assay.

Table S4. Target sequence of NCED1-1 on-target site and off-target candidate sites.

Target site	crRNA	PAM	On- or off-target sequence
NCED1-1	NCED1-1_Short	TTC	CCCAAGGCCATTGGGGAG
	NCED1-1_Middle		CCCAAGGCCATTGGGGAGCTCCAT
	NCED1-1_Long		CCCAAGGCCATTGGGGAGCTCCATGGCCAC
NCED_off-1	-	TTC	CCCAAGGCCATCGGCGAGCTCCATGGCCAC
NCED_off-2	-	TTC	CCCAAGGCCATCGGCGAGCTCCACGGCCAC

Red characters at on- or off- target sequence indicate mismatched nucleotide of off-target candidate sites. Underlines at on- or off- target sequence indicate the restriction enzyme sites for CAPS assay.

Table S5. Mutation efficiency of on- and off- target genes in regenerated T_0 plants.

crRNA	Target site	No. of regenerated T_0 plants with mutations			No. of plants
		Biallelic mutation	Monoallelic mutation	No mutation	
AAO2-1_Short	AAO2-1	18	10	8	36
	AAO_off-1	0	0	36	
	AAO_off-2	0	0	36	
	AAO_off-3	0	0	36	
AAO2-1_Long	AAO2-1	4	15	17	36
	AAO_off-1	0	0	36	
	AAO_off-2	0	0	36	
	AAO_off-3	0	0	36	
NCED1-1_Short	NCED1-1	2	3	19	24
	NCED_off-1	0	0	24	
	NCED_off-2	0	0	24	
NCED1-1_Long	NCED1-1	1	4	19	24
	NCED_off-1	0	0	24	
	NCED_off-2	0	0	24	

Table S6. The energies of the minimal free energy structures (MFE) and centroid structures.

Target site	secondary structure	energy (kcal/mol)		
		Short	Middle	Long
DL-1	MFE	-4.8	-5.4	-10.3
	Centroid	-4.8	-2.6	-10.3
DL-2	MFE	-7.4	-8.4	-21.0
	Centroid	-4.8	-0.5	-21.0
ALS-1	MFE	-4.8	-11.1	-14.2
	Centroid	-4.8	-11.1	-14.2
ALS-2	MFE	-4.8	-4.8	-4.8
	Centroid	-4.8	0.0	0.0
AAO2-1	MFE	-5.0	-5.0	-6.1
	Centroid	-2.9	-2.5	-4.8
NCED1-1	MFE	-11.4	-11.4	-17.5
	Centroid	-11.4	-11.4	-17.5

Table S7. GC contents in each crRNA.

Target site	GC contents (%)		
	Short	Middle	Long
DL-1	38	42	45
DL-2	49	53	59
ALS-1	43	47	47
ALS-2	30	26	29
AAO2-1	35	30	33
NCED1-1	46	47	51
